# A Novel Role for the RNA–Binding Protein FXR1P in Myoblasts Cell-Cycle Progression by Modulating *p21/Cdkn1a/Cip1/Waf1* mRNA Stability

**DOI:** 10.1371/journal.pgen.1003367

**Published:** 2013-03-21

**Authors:** Laetitia Davidovic, Nelly Durand, Olfa Khalfallah, Ricardo Tabet, Pascal Barbry, Bernard Mari, Sabrina Sacconi, Hervé Moine, Barbara Bardoni

**Affiliations:** 1 Institut de Pharmacologie Moléculaire et Cellulaire, CNRS UMR 7275, Valbonne, France; 2 Université de Nice-Sophia Antipolis, Nice, France; 3 IGBMC (Institut de Génétique et de Biologie Moléculaire et Cellulaire), CNRS, UMR7104, Inserm U596, Collège de France, Strasbourg University, Illkirch-Graffenstaden, France; 4 INSERM U638, Faculté de Médecine, Université de Nice Sophia-Antipolis, Centre de Référence pour les Maladies Neuromusculaires, CHU de Nice, Nice, France; The Jackson Laboratory, United States of America

## Abstract

The Fragile X-Related 1 gene (*FXR1*) is a paralog of the Fragile X Mental Retardation 1 gene (*FMR1*), whose absence causes the Fragile X syndrome, the most common form of inherited intellectual disability. FXR1P plays an important role in normal muscle development, and its absence causes muscular abnormalities in mice, frog, and zebrafish. Seven alternatively spliced *FXR1* transcripts have been identified and two of them are skeletal muscle-specific. A reduction of these isoforms is found in myoblasts from Facio-Scapulo Humeral Dystrophy (FSHD) patients. FXR1P is an RNA–binding protein involved in translational control; however, so far, no mRNA target of FXR1P has been linked to the drastic muscular phenotypes caused by its absence. In this study, gene expression profiling of C2C12 myoblasts reveals that transcripts involved in cell cycle and muscular development pathways are modulated by *Fxr1*-depletion. We observed an increase of p21—a regulator of cell-cycle progression—in *Fxr1*-knocked-down mouse C2C12 and FSHD human myoblasts. Rescue of this molecular phenotype is possible by re-expressing human FXR1P in *Fxr1*-depleted C2C12 cells. FXR1P muscle-specific isoforms bind *p21* mRNA via direct interaction with a conserved G-quadruplex located in its 3′ untranslated region. The FXR1P/G-quadruplex complex reduces the half-life of *p21* mRNA. In the absence of FXR1P, the upregulation of *p21* mRNA determines the elevated level of its protein product that affects cell-cycle progression inducing a premature cell-cycle exit and generating a pool of cells blocked at G0. Our study describes a novel role of FXR1P that has crucial implications for the understanding of its role during myogenesis and muscle development, since we show here that in its absence a reduced number of myoblasts will be available for muscle formation/regeneration, shedding new light into the pathophysiology of FSHD.

## Author Summary

Muscle development is a complex process controlled by the timely expression of genes encoding crucial regulators of the muscle cell precursors called myoblasts. We know from previous studies that inactivation of the *Fragile X related 1* (*FXR1*) gene in various animal models (mouse, frog, and zebrafish) causes muscular and cardiac abnormalities. Also, FXR1P is reduced in a human myopathy called Fascio-Scapulo Humeral Dystrophy (FSHD), suggesting its critical role in muscle that findings presented in this study contribute to elucidating. Cell-cycle arrest is a prerequisite to differentiation of myoblasts into mature myotubes, which will form the muscle. One key regulator is the p21/Cdkn1a/Cip1/Waf1 protein, which commands myoblasts to stop proliferating, and this action is particularly important during muscle regeneration. In this study, we have identified FXR1P as a novel regulator of p21 expression. We show that FXR1P absence in mouse myoblasts and FSHD-derived myopathic myoblasts increases abnormally the levels of p21, causing a premature cell cycle exit of myoblasts. Our study predicts that FXR1P absence leads to a reduced number of myoblasts available for muscle formation and regeneration. This explains the drastic effects of *FXR1* inactivation on muscle and brings a better understanding of the molecular/cellular bases of FSHD.

## Introduction

The Fragile X-Related 1 (*FXR1*) gene belongs to a small gene family that includes the Fragile X Mental Retardation 1 (*FMR1*) and Fragile X-Related 2 (*FXR2*) genes (reviewed in [1]). Human *FMR1* is located on chromosome Xq27.3 [2] and inactivation of *FMR1* expression leads to the Fragile X syndrome in human, the first cause of inherited mental retardation [5]. *FXR1* and *FXR2* are autosomal genes, respectively mapping at 3q28 and 17p13.1 [3], [4]. The *FXR1* gene is highly expressed in muscle and its pre-mRNA is known to undergo extensive alternative splicing, which generates distinct *FXR1* mRNA variants that produce FXR1P isoforms with divergent C-terminal regions [6], [7]. Four isoforms ranging from 70 to 80 KDa (Isoa, Isob, Isoc, Isod) are ubiquitously expressed, including in murine [7], [8] and human myoblasts [9]. Myoblasts also express long muscle-specific *FXR1* mRNA variants, termed Isoe and Isof, which are massively induced upon muscular differentiation [7], [8], [9], [10]. Importantly, these muscle-specific mRNA variants of *FXR1* are the only expressed in adult muscle [6], [7], [8], [9], [11]. Defects in *FXR1* gene muscular pattern of expression have been observed in patients affected by Facio-Scapulo Humeral Distrophy (FSHD), the most prevalent muscular dystrophy affecting adults and children [9]. Similar defects were observed in a mouse model of myotonic dystrophy (DM1, [12]). As a result, the long isoforms FXR1P Isoe and Isof of 82–84 kDa are depleted in myopathic muscle. Consistent with these altered expression pattern of FXR1 in myopathic patients, *Fxr1*-knockout mouse die shortly after birth most likely due to an abnormal development of cardiac and respiratory muscles [13]. A mouse model with reduced levels of *Fxr1* expression has also been generated, and displays reduced limb musculature and a shorter life span of about 18 weeks [13]. Moreover, during *Xenopus* embryogenesis, complete or partial inactivation of *xFxr1* disrupts somitic myotomal cell rotation and segmentation, impeding normal myogenesis [14]. Finally, depletion of zFxr1p during early development of the zebrafish leads to cardiomyopathy and muscular distrophy [15]. All these data point out an evolutionarily conserved role for FXR1P in myogenesis.

FXR1P contains two KH domains and one RGG box that are characteristic motifs in RNA-binding proteins [4], [16]. In addition, FXR1P harbours nuclear localization and export signals (NLS and NES) enabling nucleocytoplasmic shuttling [4], [17]. In most cell types and tissues studied, FXR1P isoforms are associated to messenger ribonucleoparticles (mRNPs) present on polyribosomes, suggesting a consensus role in translation regulation for FXR1P [18]. However, it was reported that, in undifferentiated myoblasts, FXR1P long isoforms Isoe and Isof are not detected on polyribosomes, suggesting a role other than translation regulation for these isoforms at this stage [7], [8]. Very few specific target mRNAs for FXR1P have been identified so far, and even more scarcely in the context of myogenesis. First, two independent studies reported that the shortest isoform of FXR1P, Isoa, binds the AU-rich element (ARE) present in the 3′UTR of proinflammatory cytokine tumor necrosis factor (*TNFα*) mRNA [19], [20]. In this context, FXR1P associates with AGO2 on *TNFα*−ARE to modulate its translation [20]. Second, we have previously shown the ability of FXR1P Isoe, its long muscle-specific isoform, to interact specifically and with high affinity with the G-quadruplex RNA structure *in vitro* [21]. However, no mRNA target of FXR1P bearing a G-quadruplex has been identified yet *in vivo*. Finally, one study reports the presence of *Desmoplakin* and *Talin2* mRNAs in FXR1P-mRNP complexes and subsequent disturbance of the expression of the encoded proteins in *Fxr1*-KO heart extracts [22]. However, neither the binding motif/sequence recognized by FXR1P on these mRNAs nor the exact functional significance of these interactions have been explored.

To gain further insights into the muscular roles of FXR1P and the pathways perturbed in its absence, we performed a large-scale microarray analysis of the C2C12 myoblastic cell line inactivated for *Fxr1*. This analysis revealed that *Fxr1*-depletion lead to premature cell cycle exit of myoblasts. We link this to a robust increase in the levels of the cyclin-dependant inhibitor p21/Cdkn1a/Cip1/Waf1, that is also observed in FSHD-derived myoblasts. In this study, we further explore the role played by the direct interaction of FXR1P with *p21* mRNA in the post-transcriptional control of p21 levels.

## Results

### Inactivation of *Fxr1* in C2C12 myoblasts selectively affects the expression of a range of genes associated with cell-cycle regulation during muscle development

**Figure 1:**
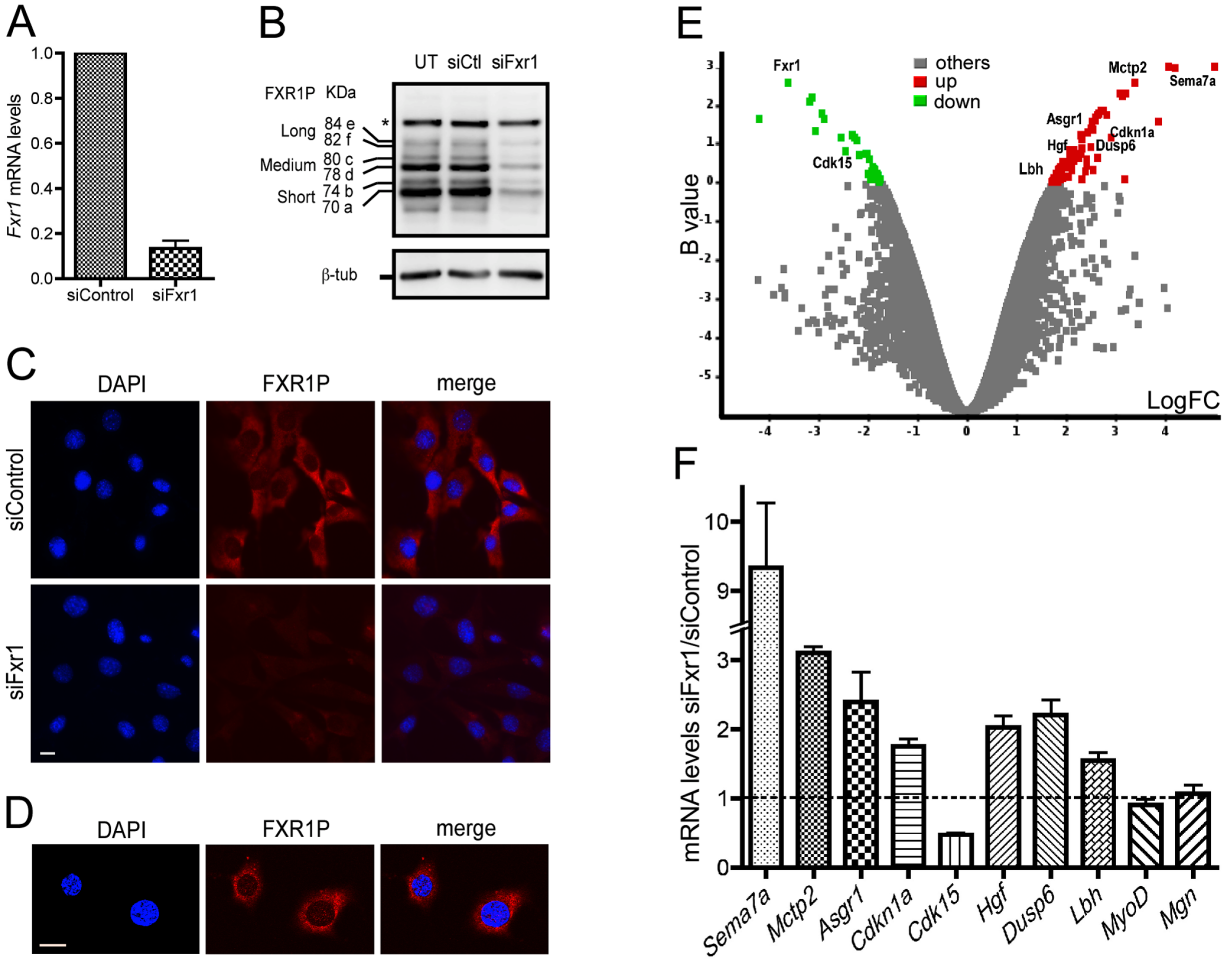
Microarray analysis of *Fxr1*-depleted C2C12 myoblasts. (A) Quantitative RT-PCR reveals a strong reduction of *Fxr1* mRNA in C2C12 cells transfected with siRNA against *Fxr1* compared to siControl-transfected cells. (B) Western-blot analysis of untransfected (UT) and siFxr1-transfected cells (siFxr1) revealed with the antibody #3FX recognizing all isoforms of FXR1P reveals a strong depletion of all isoforms of FXR1P (short, medium and long) compared to control (siCtl), while the levels of FXR2P protein (asterisk, *) remain unchanged. β-tubulin (β-tub) signal is used to verify equal loading of lanes. (C) Immunofluorescence analysis of FXR1P (red) subcellular distribution in siControl and siFxr1-transfected cells, using polyclonal #830 anti-FXR1P antibodies. Nuclei were counterstained with DAPI (blue) and merge images are shown in the right panel. The same exposure time was used for both image captures and reveal a strong depletion in FXR1P signal in siFxr1-transfected cells compared to control (siControl). Scale bar: 15 µm. (D) Confocal micrographs of C2C12 cells immunostained for FXR1P reveal a nucleocytoplasmic distribution of FXR1P. Please note the nuclear dot-like structures containing FXR1P. Slice depth: 1 µm, scale bar: 15 µm. (E) Volcano plot showing the distribution of differentially expressed transcripts between C2C12 cells transfected with siRNA against *Fxr1* versus siControl-transfected cells. Log of the fold of change (LogFC) is plotted against the B-statistic value for each transcript. A subset of 9 transcripts selected for further validation by Quantitative-RT PCR (*Fxr1*, *Cdk15*, *Sema7a*, *Mctp2*, *Asgr1*, *Hgf*, *p21*, *Dusp6* and *Lbh*) are highlighted. Significantly down- and up-regulated genes are shown in green and red, respectively. (F) Quantitative-RT PCR analysis of a subset of mRNAs confirm that *Sema7a*, *Mctp2*, *Asgr1*, *p21*, *Hgf*, *Dusp6*, *Lbh*, *MyoD* and *Myog* are significantly upregulated in *Fxr1*-depleted C2C12 myoblast, while *Cdk15* is downregulated, confirming the microarray analysis. Data are presented as means ± SEM of n = 4 experiments.

To understand the functional role of FXR1P in myoblasts, we used as a cellular model the C2C12 myoblastic cell line. This murine cell line enables to reproduce myogenesis *in vitro* [23] and expresses all the myogenic factors as well as FXR1P [7], [8]. In this model, we inactivated the expression of all FXR1P isoforms by transient transfection of siRNAs targeting exon 14, a constitutive exon present in all *Fxr1* mRNAs [6]. As shown in Figure 1A, quantitative RT-PCR performed on C2C12 cells transfected with siFxr1 siRNAs reveals a significant reduction in *Fxr1* mRNA as compared to siControl-transfected cells (13.45%±3.4% residual expression, Figure 1A). Knockdown of all isoforms of FXR1P was obtained by siFxr1 transfection, as shown by western-blot analysis using the 3FX antibody (Figure 1B, [8]). Note that the levels of FXR2P, the close homologue of FXR1P, also recognized by 3FX antibody, remain unaffected, confirming the specificity of the knockdown strategy (asterisk, Figure 1B). In siFxr1-transfected myoblasts, the decrease in epifluorescence signal after FXR1P-immunolabeling as compared to siControl-transfected cells confirms the efficiency of the knockdown (Figure 1C). The knockdown appears to homogenously affect all the cells since the signal is uniformly decreased. Note that in C2C12 cells, FXR1P immunoreactivity is mainly cytoplasmic, however, signal is also detected in the nucleus (Figure 1C). Indeed, we confirmed the partial nuclear localization of FXR1P in myoblasts by confocal microscopy (Figure 1D), as described previously for the long isoforms of FXR1P in C2C12 myoblasts [7] and in human myoblasts [9].

To determine the impact of the inactivation of *Fxr1* on gene expression in myoblasts, total RNA was extracted from siControl and siFxr1-transfected C2C12 myoblasts and simultaneously analysed using whole genome mouse microarrays. Among the genes showing measurable differential levels of expression, a significant change was observed for 105 transcripts (32 down- and 73 up-regulated) of which 79 were annotated in the RefSeq database (Figure 1E and Table S1). As expected, *Fxr1* mRNA appears among the most significantly down-regulated in siFxr1-transfected cells (Figure 1E and Table S1). To confirm the observed dysregulation of a subset of mRNAs in *Fxr1*-knockdown C2C12 myoblasts, we performed quantitative RT-PCR analysis (Figure 1F). Interestingly, in *Fxr1-*depleted myoblasts, we were able to confirm by quantitative RT-PCR a significant upregulation of mRNAs encoding: Semaphorin 7a (*Sema7a*), the Ca^2+^-binding multiple C2 domains transmembrane protein 2 (*Mctp2*), asialoglycoprotein receptor 1 (*Asgr1*), the cyclin-dependant kinase inhibitor p21 (*p21/Cdkn1a/Waf1/Cip1*), Hepatocyte growth factor (*Hgf*), Dual specific phosphatase (*Dusp6*) and finally Limb-bud and heart protein (*Lbh*, Figure 1E). Conversely, we confirmed a significant down-regulation of *Cdk15* mRNA encoding the cyclin-dependent kinase 15. Finally, the mRNAs encoding the myoregulatory factors MyoD and Myogenin for which no mRNA variations were detected by microarray analysis remained unaffected (Figure 1F). These analyses were further repeated on C2C12 cells inactivated for *Fxr1* by transfection of a different siRNA (siFxr1#2) targeting *Fxr1* exon 6, another constitutive exon of *Fxr1* present in all its variants [6]. This second siRNA leads to a 37% residual expression of *Fxr1* mRNA (Figure S1A) and reduces all FXR1P isoforms (Figure S1B) as compared to siControl. In addition, siFxr1#2-mediated knockdown of *Fxr1* efficiently modulated the previously studied subset of mRNAs to induce variations similar to the one observed with the first siRNA against *Fxr1* (Figure S1C). Importantly, this cross-analysis using two siRNAs targeting distinct regions of *Fxr1* mRNA exclude the fact that the observed variations could derive from off-target effects of the siRNAs.

To gain insights into the pathways perturbed by *Fxr1* depletion, we performed an analysis of the biological functions or processes selectively enriched among the altered transcripts, using the Ingenuity Pathway Analysis (IPA) software (Table S2). Interestingly, *Fxr1* knockdown in C2C12 myoblasts significantly affected the functional categories ‘cell cycle’ (Table S2), ‘skeletal and muscular system development and function’ and ‘skeletal and muscular disorders’ (Table S2). Importantly enough, a subset of mRNAs perturbed in siFxr1-knockdown myoblasts compared to control repeatedly appeared determinant for the definition of the affected functional categories: the cyclin-dependent kinase (*Cdk15*), the cyclin-dependent kinase inhibitor (*p21/Cdkn1a/Cip1/Waf1*) and the Hepatocyte growth factor (*Hgf*).

### *Fxr1-*depletion in myoblasts leads to premature exit of cell cycle

**Figure 2:**
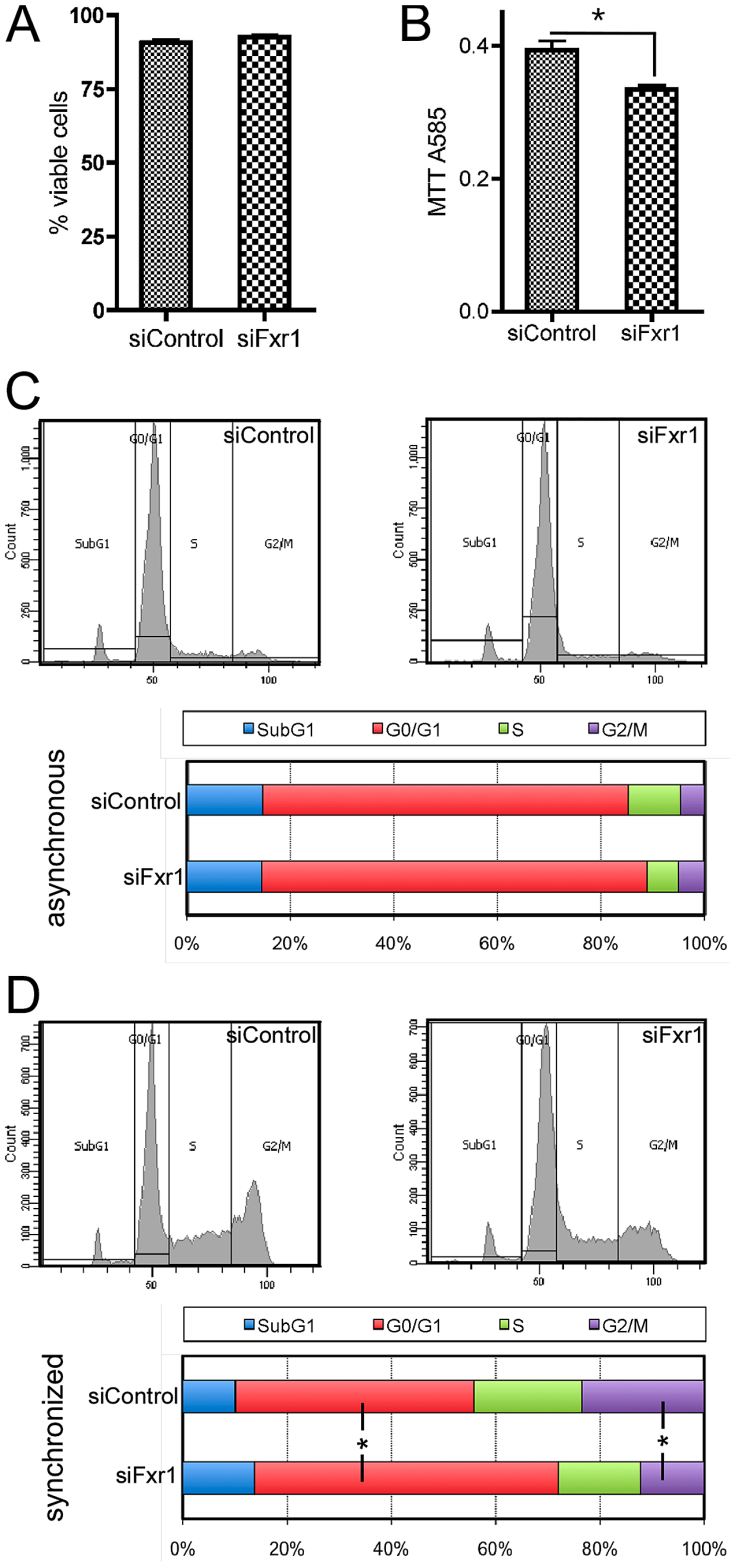
*Fxr1*-depletion does not impair myoblasts viability but specifically induces accumulation in G0/G1 phase to the detriment of mitosis. (A) PI incorporation in living siFxr1- or siControl-transfected cultures and subsequent FACS analysis was performed to show that viability of the culture is not affected by *Fxr1*-depletion. (B) MTT colorimetric assay show that the proliferation abilities of C2C12 cells are significantly impaired by *Fxr1*-depletion. (C) FACS analysis of the Propidium Iodure-stained DNA content of C2C12 cells transfected with siControl or siFxr1. Cells were analysed in asynchronous conditions or following synchronisation treatment for 8 hrs with the cell cycle blocker mimosine (late G1) followed by 16 hrs release in normal growth medium (D). In asynchronous conditions, cell cycle distribution is similar in siControl or siFxr1 transfected cells. Synchronisation of cells allows detecting significant differences in the distribution of the cells in the various cell cycle absence of FXR1P: increase in the G0/G1 proportion and decrease in the G2/M. Data are presented as means ± SEM of n = 4 experiments, with FACS analysis of a minimal cell population of 15,000 for each condition and each experiment. The asterisk (*) indicates p<0.05 of a Mann & Whitney test.

One of the most recurrent terms in IPA analysis of dysregulated mRNA upon *Fxr1* depletion were ‘cell cycle progression’, ‘arrest in G0/G1’, ‘proliferation’ and also ‘cell viability’ (Table S2). This prompted us to analyse myoblasts' viability and proliferation abilities upon *Fxr1-*depletion. Fluorescence-Activated Cell Sorting (FACS) analysis of the DNA intercalant Propidium Iodide (PI) incorporation on living cells allowed us to detect no changes in the overall viability of *Fxr1*-knockdown (92.5% viability) compared to control (90.53% viability) C2C12 cells (Figure 2A). To assess the proliferation ability of *Fxr1*-depleted myoblasts, we conducted tetrazole MTT proliferation assays. Interestingly, after 48 hours in culture, siFxr1-transfected C2C12 cells exhibit a significant 15% decrease in MTT reductase activity as compared to control (Figure 2B). This suggests that *Fxr1* depletion may induce alterations of myoblasts cell cycle. We therefore further analysed the distribution in the various cell cycle phases of siFxr1- or siControl transfected myoblasts. The DNA content of the cells was assessed by FACS-measurement of the amount of PI incorporated in cells. Surprisingly, in a normal asynchronous cell population, we did not observe any significant change in the cell cycle phases distribution of the C2C12 cells transfected with siFxr1 or siControl, in normal growth conditions (Figure 2C).

To highlight specific defects in cell cycle, we synchronized siFxr1- and siControl-transfected myoblasts by treatment with the cell cycle blocker mimosine, that arrests cell cycle progression at the G1/S phase border [24]. Since the effects of this cell cycle blocker are fully reversible, we then allowed the synchronized cells to reenter cell cycle by incubating them in normal growth medium for 16 hrs before FACS analysis. In these conditions, we did observe a significant 27.6% increase in the number of cells in the G0/G1 phase in *Fxr1*-knockdown myoblasts, as compared to control. This increase in the G0/G1 population is accompanied by a 51.9% decrease in the number of cells in the G2/M phase. Importantly, no differences were observed in the proportion of cells in the Sub-G1 phase - corresponding to cellular debris with a lower DNA content liberated by apoptotic cells [25]- in asynchronous cells (Figure 2A) and after release from cell cycle blocker (Figure 2D). These data indicate that FXR1P depletion in myoblasts does not lead to cell viability defects but rather causes a blockade and accumulation of cells in the G0/G1 phase to the detriment of mitosis.

**Figure 3:**
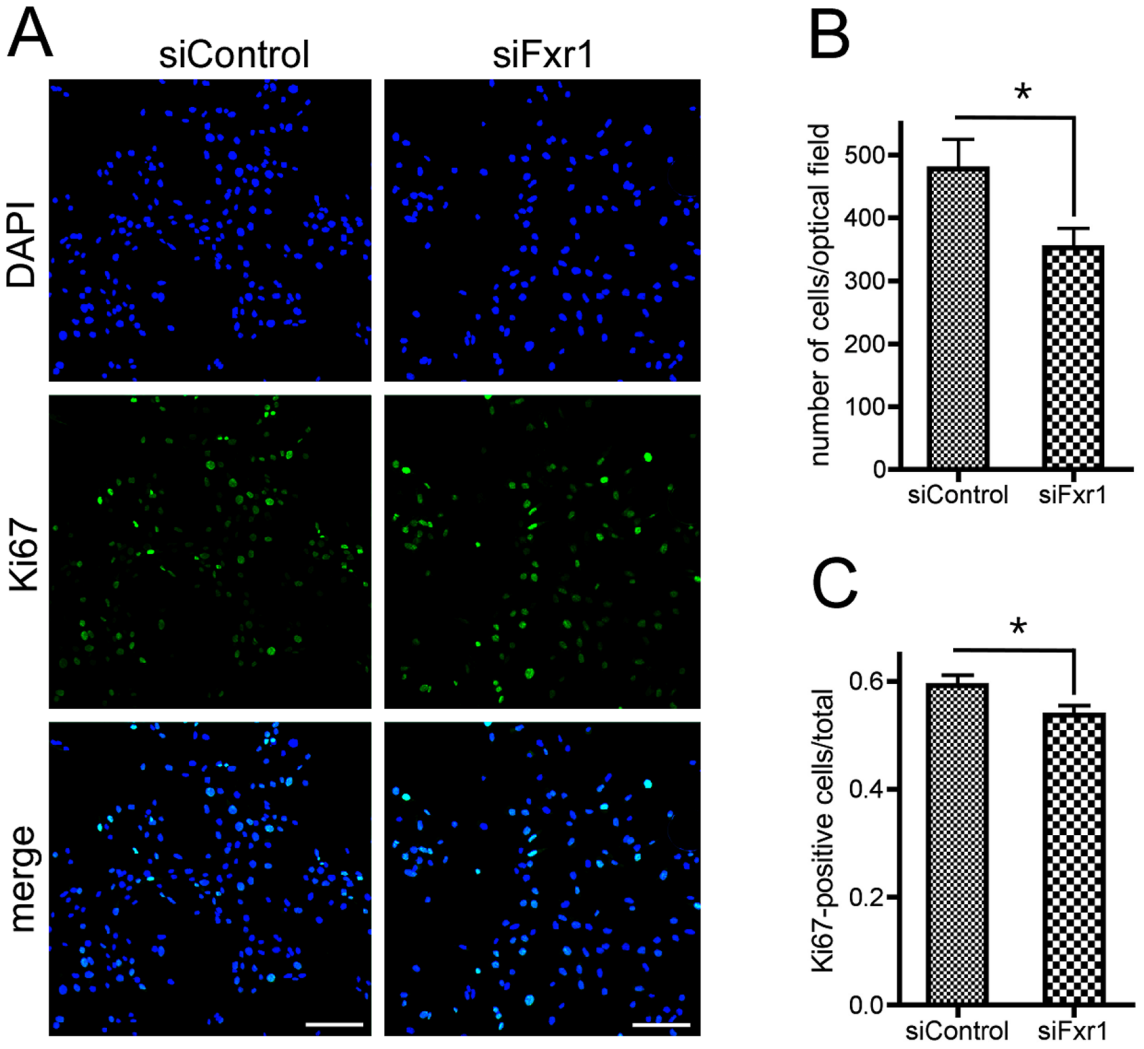
Knockdown of FXR1P induces premature cell cycle exit of myoblasts. (A) Immunofluorescence analysis of C2C12 cells transfected with siControl or siFxr1. Nuclei are stained with DAPI (blue) and cells expressing the proliferation marker Ki67 are labelled with FITC antibody (green). Scale bar: 75 µm. (B) Quantification of the number of DAPI-stained nuclei. (C) Quantification of the number of Ki67-positive cells over total number of nuclei quantified in (B). Quantification was performed using a macro developed with the ImageJ software. Data presented are mean of n = 4 experiments with analysis of 10 optical fields for each condition and each experiment. The asterisk (*) indicates p<0.05 for the Student T-test.

Thus, to determine whether the cells were blocked in G0 or G1, we performed immunolabeling of C2C12 cells in normal growth conditions and quantified the number of DAPI-positive nuclei and the amount of cells positive for the proliferation marker Ki67 (Figure 3). We observed that the number of nuclei in cultures of siFxr1-transfected myoblasts is decreased by 26%, suggesting that *Fxr1* depletion limits the proliferating abilities of myoblasts (Figure 3B). Quantification of cells expressing Ki67 enabled us to detect that siRNA-meditated depletion in *Fxr1* leads to a subtle, but significant 10% decrease in the number of Ki67-positive cells compared to control (Figure 3C). Since Ki67 is expressed during all active phases of the cell cycle (G1, S, G2, and mitosis), but absent from quiescent cells (G0) [26], the unlabeled cells most likely represent resting cells blocked in G0.

### The absence of FXR1P in C2C12 cells and in FSHD patients-derived myoblasts affects the levels of endogenous *p21* mRNA and protein

The premature cell cycle arrest we observed in *Fxr1-*depleted myoblasts prompted us to examine the subset of deregulated mRNAs identified by microarray analysis in order to identify candidates for regulation by FXR1P that could contribute to explain this phenotype. The most promising mRNA candidate appeared to encode the ubiquitous cyclin-dependent kinase inhibitor (CDKI) p21 –also known as Cdkn1a/Cip1/Waf1- that belongs to the Cip/Kip family of CDKI. In myoblasts, p21 is known to block cell cycle progression to trigger cell-cycle exit, a prerequisite to muscular differentiation [27], [28], [29].

**Figure 4:**
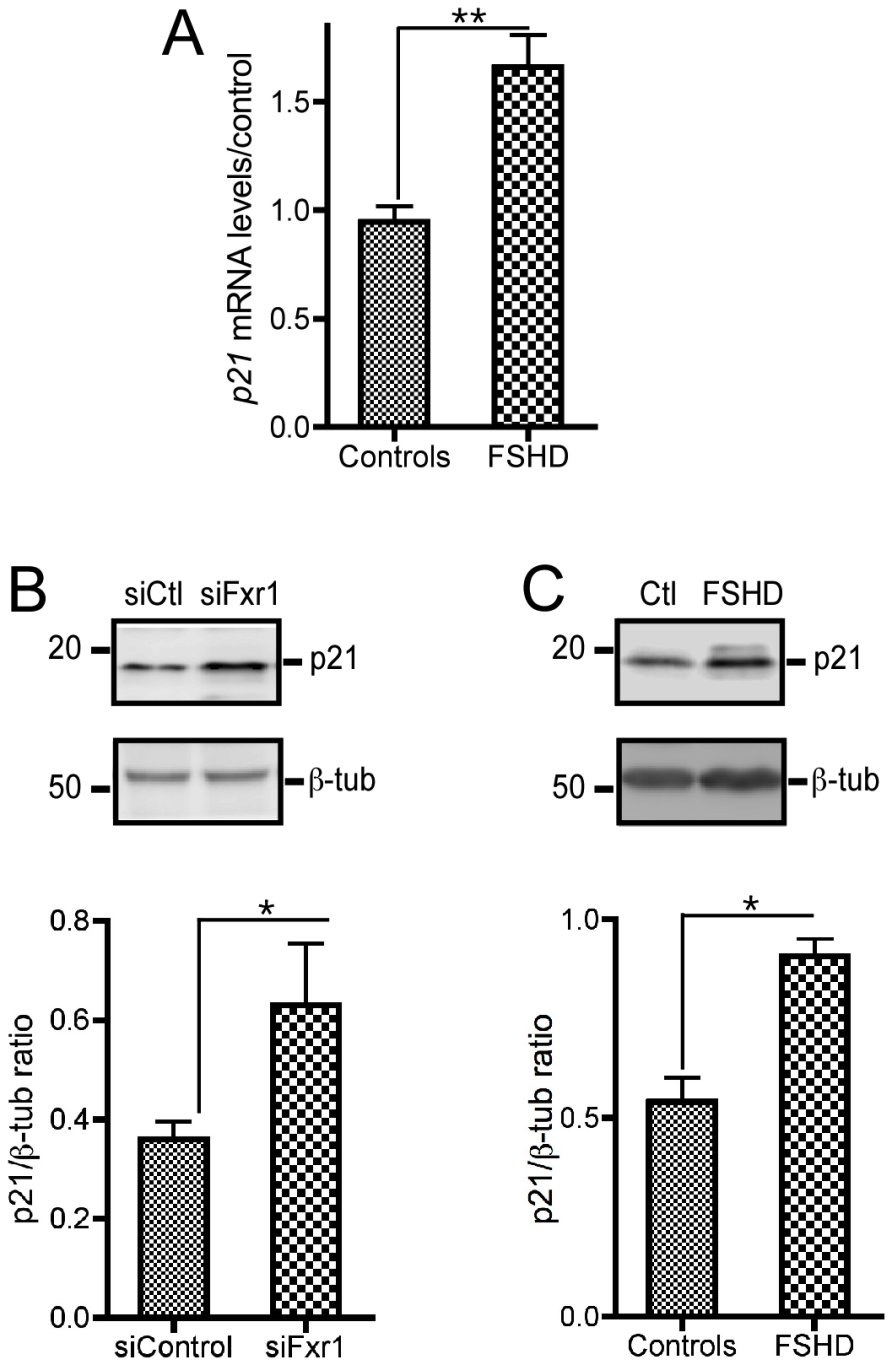
FXR1P depletion in C2C12 cells and in myoblasts derived from FSHD myopathic patients biopsies contributes to a consistent increase in *p21* mRNA that translates into enhanced p21 protein levels. (A) Quantitative RT-PCR reveals a significant increase of *P21* mRNA in FSHD myoblats relative to control individuals. Data are presented as means ± SEM of n = 3 individuals/group. (B) Representative western-blot of p21 protein levels in siControl (siC) or siFxr1 (siFx)-transfected C2C12 cells. Densitometric quantification of western-blots reveal that depletion of FXR1P by siRNA transfection (siFxr1) leads to a significant increase of p21 protein levels relative to siControl-transfected cells. Data are presented as means ± SEM of n = 4 experiments. (C) Representative western-blot of P21 protein levels in FSHD patients and control individuals. Densitometric quantification of western-blots reveals that muscle biopsies of FSHD patients display a significant increase of P21 protein relative to controls. Data are presented as means ± SEM of n = 3 individuals/group. The asterisks * and ** indicate respectively p<0.05 and p<0.01 of the Mann & Whitney test.

In *Fxr1-*depleted myoblasts, we found that *p21* mRNA level is significantly increased by microarray analysis (Figure 1E, Table S1) and confirmed a 1.76-fold upregulation of the transcript by quantitative-RT PCR in these *Fxr1* loss-of-function experiments (cf Figure 1F). This upregulation of *p21* mRNA level in *Fxr1*-depleted myobasts was further confirmed using a second siRNA targeting *Fxr1* (Figure S1). We had previously shown that the muscle-specific long isoforms of FXR1P, notably Isoe, are depleted in myoblasts derived from Fascio-ScapuloHumeral Distrophy (FSHD) patients and had hypothesized that this could induce deregulation of mRNA targets specific to this isoform FXR1P Isoe [9]. To test this hypothesis on this new potential mRNA target of FXR1P, we assessed the status of human P21 in the same samples used in our previous study. Interestingly enough, *P21* mRNA levels are significantly increased in FSHD patients by a 1.8 factor (Figure 4A).

We then sought to verify whether this increase in *p21* at the mRNA level was translated at the protein level by western-blotting (WB) analysis. Quantification of WB of siFxr1-transfected C2C12 using the ImageJ software revealed a 1.92 fold increase in p21 protein levels (Figure 4B). Concomitantly, we observed by western-blotting that the levels of P21 protein are increased in FSHD myoblasts compared to control by a 1.66 factor (Figure 4C). These data indicate that depletion of FXR1P and particularly of its long muscle-specific isoforms increases *p21* mRNA and correlatively increase the levels of p21 protein both in murine and human myoblasts.

**Figure 5:**
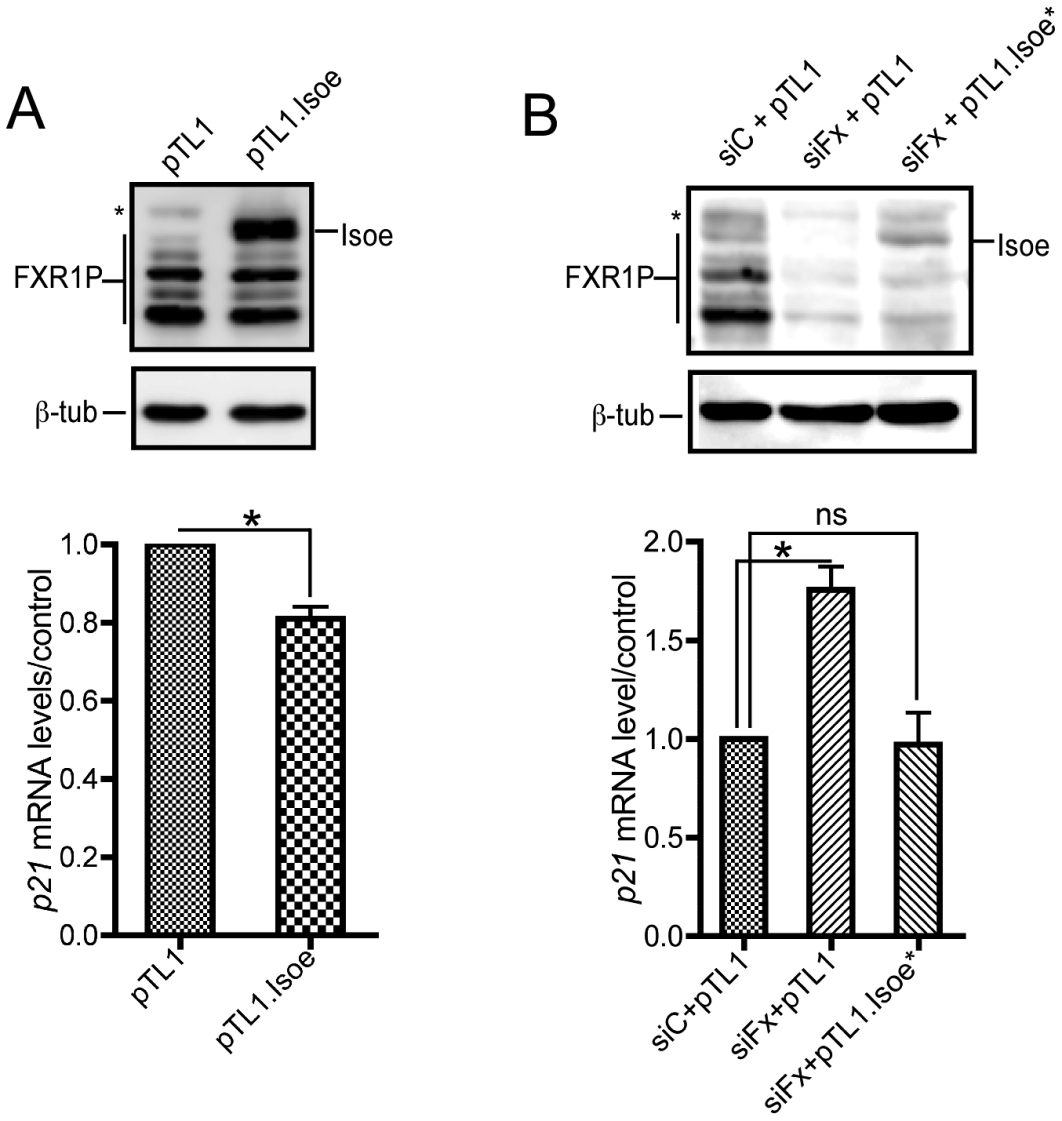
FXR1P overexpression in *Fxr1*-depleted C2C12 cells restores *p21* mRNA levels to normal. (A) Western-blot analysis (upper panel) of C2C12 cells transfected with empty pTL1 vector or pTL1.FXR1 Isoe (pTL1.Isoe) construct indicate a strong expression of FXR1P long isoform Isoe in transfected myoblasts. Quantitative RT-PCR (lower panel) reveals a significant decrease of *p21* mRNA levels in C2C12 myoblasts overexpressing FXR1 Isoe, as compared to control. Data are presented as means ± SEM of n = 3 independent experiments. (B) Western-blot analysis (upper panel) of C2C12 cells transfected with control siRNA (siC) or siFxr1 (siFx) and empty pTL1 vector or a mutated version of pTL1.FXR1 Isoe (pTL1.Isoe*) bearing 4 mismatches in siFxr1 recognition sequence indicate a reexpression of FXR1P long isoform Isoe in *Fxr1*-depleted transfected myoblasts. In the western blot FXR2P is indicated by (*) Quantitative RT-PCR (lower panel) reveals a significant increase of *p21* mRNA levels in C2C12 myoblasts transfected with siFxr1 (siFx) and the empty vector (pTL1), as compared to control. This increase is restored to normal levels when FXR1P Isoe expression is rescued by transfection of pTL1.FXR1 Isoe. Data are presented as means ± SEM of n = 3 independent experiments. The asterisks * indicate p<0.05 of the Wilcoxon paired test, ns indicates non significance.

To assess the specificity and the direct nature of the effects we observed on p21 mRNA levels by FXR1P loss of function experiments, we first used a gain-of-function approach. For these experiments, we used FXR1P long isoform Isoe since its depletion in FSHD myoblasts recapitulates the effects on *p21* mRNA levels of a knockdown of all FXR1P isoforms in C2C12 cells (cf Figure 4). Interestingly, in contrast to *Fxr1* loss-of-function in C2C12 myoblasts, over expression of FXR1P Isoe lead to a 19,1% significant decrease in endogenous *p21* mRNA levels as compared to transfection with empty vector (Figure 5A). This ascertains the fact that the effects we observe on *p21* mRNA levels are directly related to the levels of FXR1P present in the cell. Secondly, we performed rescue experiments using a pTL1 plasmid bearing *FXR1* Isoe cDNA in which we generated by site-directed mutagenesis 4 mismatches to avoid recognition of the transgene by siFxr1 (Figure 5B). This strategy enabled to efficiently re-express FXR1P Isoe in *Fxr1*-knocked down myoblasts (Figure 5C). Rescue of the expression of FXR1P Isoe lead to a significant reduction in *p21* mRNA levels as compared to unrescued myoblasts. The rescue with FXR1P Isoe is total since the levels of *p21* mRNA in rescued cells are restored to control levels. Of notice, similar results were obtained using another mutant plasmid of pTL1.Isoe (data not shown), confirming the efficiency of the rescue strategy.

These data confirm the specificity of our approach and suggests that *p21* mRNA may be a target of FXR1P in C2C12 murine myoblasts and in human myoblasts, either directly by RNA-protein physical interaction, or indirectly by modulating a pathway involved in *p21* levels controls.

### *p21* mRNA is a novel mRNA target of FXR1P, both *in vitro* and *in vivo*

**Figure 6:**
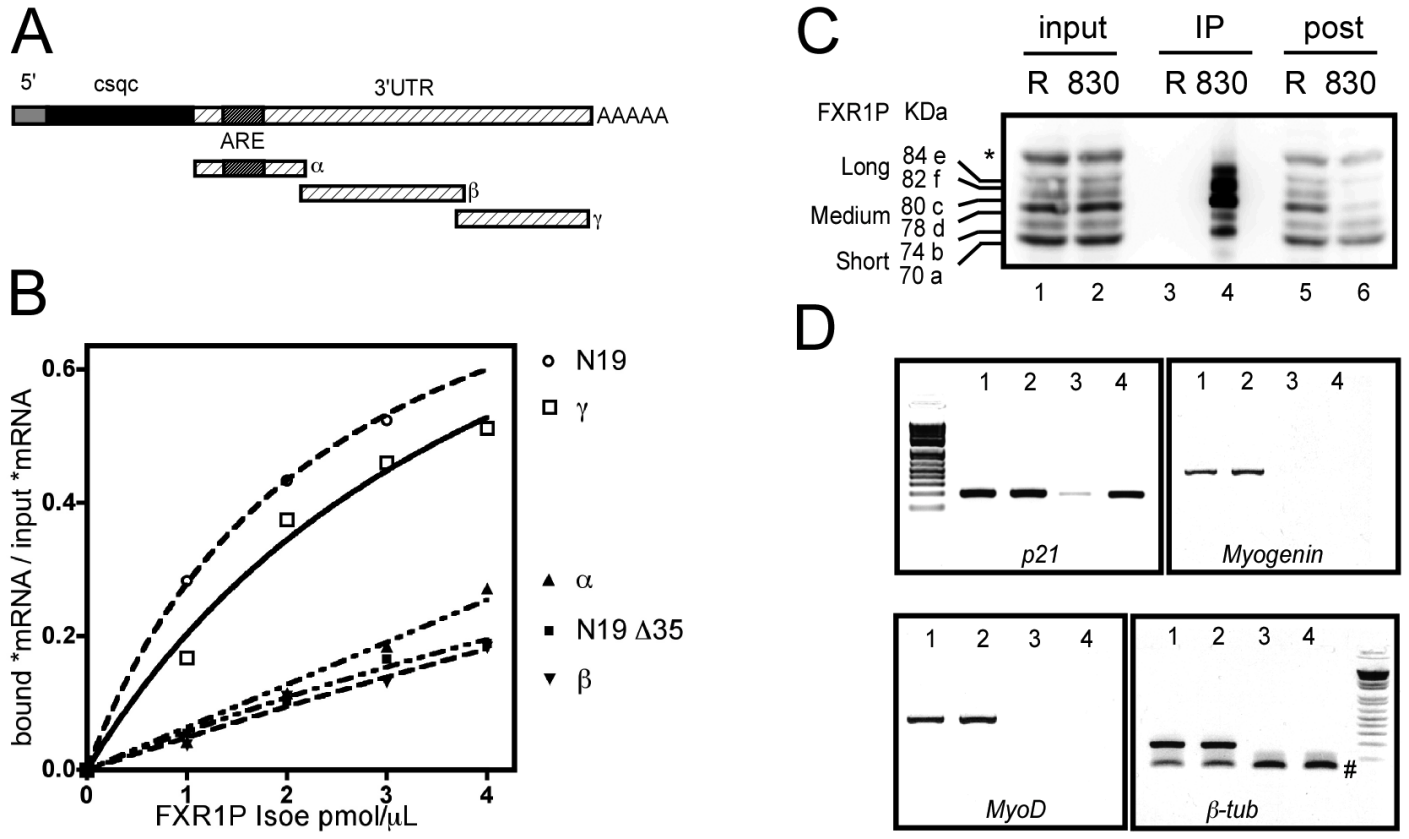
FXR1P selectively binds *in vitro* to the distal portion of *p21* mRNA 3′ UTR and associates *in vivo* with *p21* mRNA. (A) Scheme of the various portions of *p21* mRNA 3′ UTR (α, β and γ) used for *in vitro* binding assays. Note that the α fragment contains a characterized ARE motif. (B) Nitrocellulose filter binding assays to determine the portion of *p21* mRNA bound by FXR1P. Radiolabeled mRNA probes were incubated with increasing concentrations of recombinant FXR1P Isoe protein, the amount of radioactive probes recovered on filters after binding reaction is then plotted against the concentration of proteins. The portion of *FMR1* mRNA called N19 (known to be bound by FXR1P) and its truncated version (N19Δ35) were used as controls. This reveals that the distal portion of *p21* 3′UTR (γ fragment) and N19 are selectively bound by FXR1P. Both the α and β fragments from the 3′UTR of *p21* remain at background levels comparable to N19Δ35 binding to FXR1P. (C) Western-blot analysis of UV-crosslinking and immunoprecipitation (CLIP) assay performed on C2C12 lysates using polyclonal antibodies raised against the C-terminus of FXR1P (#830) and control rabbit IgG (R). Input lysates (lanes 1 & 2, Input, 1/50^th^), immunoprecipitates (lanes 3 & 4, IP, 1/5^th^) and post-immunoprecipitation supernatants (lanes 5 & 6, post, 1/50^th^) were probed for FXR1P using the 3FX antibody. A selective enrichment in FXR1P medium and long isoforms is observed in #830 immunoprecipitate (lane 3), concomitant with a depletion in these isoforms in the post-immunoprecipitation supernatant (lane 4) as compared to corresponding controls (lane 3 & 5). (D) RT-PCR analysis of mRNAs associated with FXR1P complexes. RNA was extracted from input and immunoprecipitate fractions described in (C), and used as template for RT-PCR. RT-PCR products obtained from inputs and immunoprecipitations respectively from control with rabbit IgG (Lanes 1, 3) and immunoprecipitation of FXR1P using #830 (Lanes 2, 4) were separated and visualized by agarose gel electrophoresis. This reveals that *p21* mRNA is selectively enriched in the #830 immunoprecipitates, while the mRNAs encoding the myogenic determination factors Myogenin and MyoD or the unrelated mRNA encoding β-tubulin are not recovered in any immunoprecipitates. The symbol # indicates aspecific PCR products corresponding to *β-tub* primers dimers. DNA molecular weight markers presented on the gels are respectively 100, 200, 300, 400, 500, 600, 800 and 1000 bp.

Murine *p21* mRNA is 1910 nts long (GenBank Accession number: GI 161760647), with a very short 5′UTR of less than 100 nts, a 480 nts coding sequence and a 1329 nts long 3′UTR where lie most of the regulatory elements for the stability of this mRNA (Figure 6A). Notably, the ARE located at position 86–103 nts on the 3′UTR is bound by the RNA-binding protein HuR to regulate the stability of the mRNA during muscle differentiation [30]. Given the ability of FXR1P Isoa to bind ARE sequences [19], [20], we hypothesized that the ARE present in *p21* mRNA could be the binding site of FXR1P.

To test the physical interaction between FXR1P and *p21* mRNA and determine the portion of the mRNA involved in the interaction, we performed *in vitro* filter-binding assays [21] using recombinant FXR1P and radiolabeled fragments of *p21* mRNA 3′UTR described in Figure 5A. We chose to use FXR1P Isoe, the longest muscle-specific isoform of FXR1P for binding experiments since i) it was described to have RNA-binding properties [21], ii) its depletion in FSHD myoblasts recapitulates the effect on *p21* mRNA levels of a complete knockdown of all FXR1P isoforms in C2C12 cells (cf Figure 4) and iii) Isoe is able to restore *p21* mRNA levels to normal in *Fxr1*-knockdown myoblasts (cf Figure 5). As controls for interaction, we used the N19 fragment of *FMR1* mRNA containing a G-quadruplex RNA structure [31], known to be specifically bound by FXR1P Isoe, and its truncated version N19Δ35 unable to be bound by FXR1P [21]. As expected, FXR1P was able to recognize the G-quadruplex containing N19 fragment (Figure 6B). Surprisingly, the binding activity of FXR1P towards *p21* 3′UTR-α fragment (nts 1–345) that contains a well characterized ARE sequence was null, being equal to the binding activity of the negative control N19Δ35. Also, *p21* 3′UTR-β fragment (nts 324–868) was not recognized by FXR1P. Interestingly, the most distal portion of *p21* 3′UTR, termed γ fragment (nts 851–1321), was specifically bound by FXR1P. These data indicate that FXR1P Isoe does not recognize *p21* mRNA *via* the ARE motif present in the proximal portion of the 3′UTR (α fragment), but most likely via an uncharacterized motif or sequence present in the distal portion of its 3′UTR-γ fragment.

Knowing that FXR1P interacted, at least *in vitro*, with *p21* mRNA, we further sought to validate that this interaction occurs *in vivo*. To test this hypothesis, we isolated immunocomplexes containing FXR1P by performing UV-crosslinking and immunoprecipitation assays (CLIP, [32]). Immunoprecipitation of FXR1P mRNA complexes was carried out using the polyclonal antibody #830 against exon 16 of FXR1P present in all isoforms except the short ones [7], [8] on C2C12 cell extracts (Figure 6C). Control CLIP was performed using non-immune rabbit IgGs. As expected, using the #3FX monoclonal antibody [7] against the constitutive exon 14 present in all isoforms of FXR1P, all the isoforms of FXR1P were detected in both inputs (Figure 6C, lane 1 and 2). Medium and long isoforms of FXR1P were selectively enriched in #830 immunoprecipitates (Figure 6C, Lane 4) and concomitantly depleted in #830 post-immunoprecipitation supernatant (Figure 6C, lane 6). The low amount of FXR1P small isoforms detected in the #830 immunoprecipitates most likely corresponds to the fraction of small isoforms interacting with FXR1P medium and long isoforms, since FXR1P is known to homodimerize [4]. In contrast, FXR1P is not recovered in immunoprecipitates obtained with control rabbit IgGs (Figure 6C, lane 3) and still present in the corresponding post-immunoprecipitation supernatant (Figure 6C, lane 5), confirming the specificity of the CLIP assay performed with #830 antibodies.

RT-PCR analysis of mRNAs extracted from both inputs and immunoprecipitates was then carried out (Figure 6D). The mRNA encoding p21, β-tubulin and the myogenic factors Myogenin and MyoD are detected in the input fractions (Figure 6D, lanes 1 and 2). Interestingly, only *p21* mRNA was found selectively enriched in #830 immunoprecipitates (Figure 6D, lane 4) as compared to control immunoprecipitates (Figure 6D, lane 3), while *Myogenin*, *MyoD* and *β-tubulin* mRNAs were undetectable. This confirms the specificity of the approach and suggests that, in the C2C12 myoblastic cell line, endogenous *p21* mRNA is present in mRNA complexes containing FXR1P.

### The γ fragment of p21 3′UTR recognized by FXR1P has intrinsic stabilization properties

**Figure 7:**
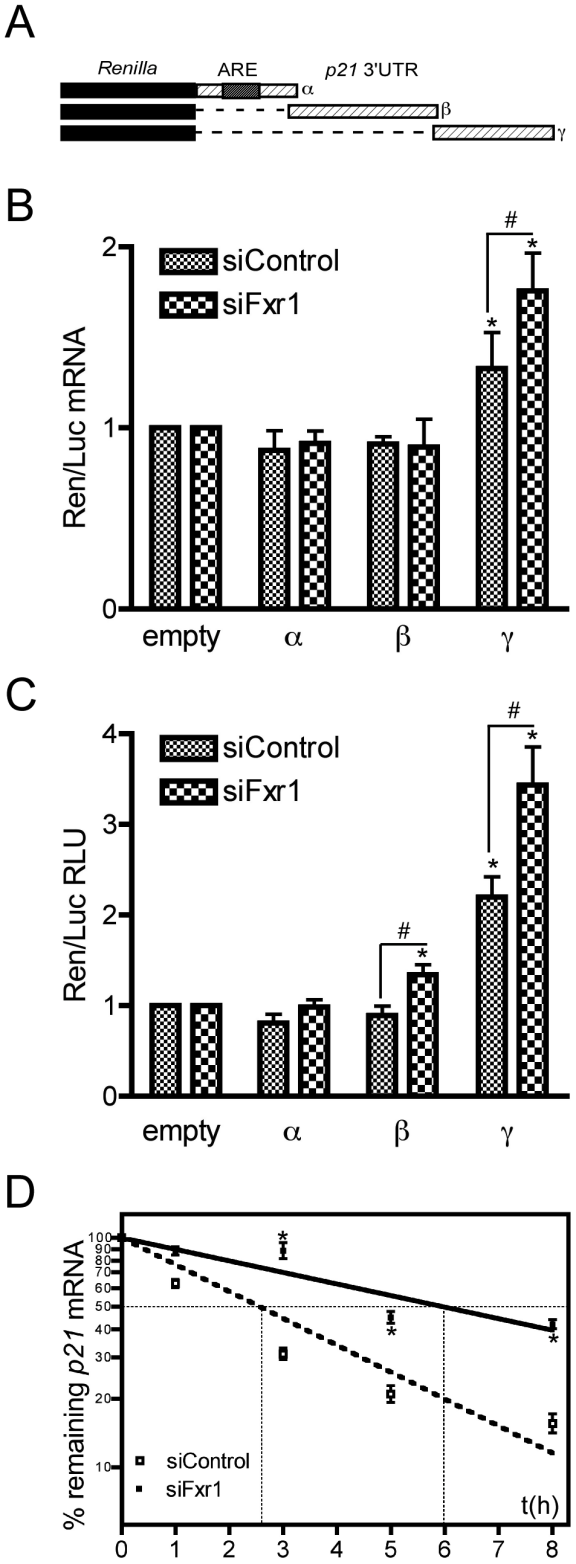
The γ portion of *p21* mRNA 3′UTR modulates the stability of the mRNA that is potentiated by FXR1P depletion. (A) Scheme of the constructs bearing various portions of *p21* mRNA 3′UTR (α, β and γ) used for luciferase assays. (B) Effect of *p21* 3′UTR−α, −β and −γ fragments on *Renilla luciferase* (*Ren*) mRNA levels in C2C12 cells transfected with control siRNAs (siControl) or siRNAs targeting *Fxr1* (siFxr1). Quantitative RT-PCR analysis of the levels of *Ren* mRNA normalised to *Firefly* (*Luc*) mRNA relative to the empty construct are presented. In siControl cells, only the γ fragment significantly increased *Ren* mRNA levels, this effect is potentiated by *Fxr1* depletion with siFxr1. In contrast, the α and β fragment have no effect on *Ren* mRNA levels, in the presence or absence of FXR1P. The results are presented as the means ±SEM of 4 experiments. (C) Effect of *p21* 3′UTR and its α, β and γ fragments on Renilla Luciferase activity in C2C12 cells transfected with control siRNAs (siControl) or siRNAs targeting *Fxr1* (siFxr1). Results presented here represent the mean of the ratio of Luc-FL, Luc-α, Luc-β and Luc-γ to Luc-empty signal. In siControl cells, only the γ fragment significantly increased luciferase activity. In siFxr1 transfected cells compared to controls, the β and γ fragments increased luciferase activity, while the α fragment has no effect. However, the amplitude of variation is greater with the γ fragment and this effect is potentiated by *Fxr1* depletion. Six independent experiments in triplicate for each transfection were quantified. For each transfection, Renilla was normalized to Firefly luciferase activity. RLU, relative luciferase units. (D) *Fxr1*-depletion increases the stability of endogenous *p21* mRNA. C2C12 transfected with siControl (empty squares) or siFxr1 (black squares) were treated with the transcription inhibitor actinomycin D for 8 hrs. *p21* mRNA levels were determined by quantitative RT-PCR at several time points and normalised to levels before treatment (t0). Percentage of remaining mRNA is plotted using a semi-log scale. Data presented represent the mean of n = 3 experiments. The asterisks * indicate p<0.05 of the Mann & Whitney test, while # and ## indicate respectively p<0.05 and p<0.01 of the Wilcoxon test.

To elucidate the functional significance of FXR1P interaction with *p21* 3′UTR-γ fragment, we conducted luciferase assays on C2C12 cells expressing FXR1P normally (siControl-transfected) and inactivated for *Fxr1* (siFxr1-transfected). The various portions of *p21* 3′UTR used for binding assays were cloned in the 3′ of *Renilla* luciferase cDNA in a reporter system (Figure 7A). The influence of the 3′ regulatory elements on *Renilla* mRNA and protein levels was then assessed, in the presence and in the absence of FXR1P, and compared to control vector without regulatory elements in the 3′UTR (Figure 7B, 7C). In the presence of FXR1P or when FXR1P is knocked-down, no significant difference to control is observed in the *Renilla* mRNA levels, when its cDNA is fused either to the proximal α or central β fragments of *p21* mRNA 3′UTR. However, the distal γfragment bound by FXR1P significantly increases *Renilla* mRNA levels in the presence of FXR1P (1.33-fold, Figure 7B). Intriguingly, removal of FXR1P by siRNA-mediated knockdown potentiated the mRNA stabilizing effect of the *p21* 3′UTR-γ fragment (1.76-fold; Figure 7B) compared to control. To assess whether variations of *Renilla* mRNA correlated to protein variations, we performed classical luminescence luciferase assays (Figure 7C). Interestingly, *Fxr1*-depletion lead to a significant increase in Renilla luciferase activity when its cDNA was either fused to the central β or distal γ fragment of *p21* 3′UTR (Figure 7C). However, the amplitude of variation was, again, higher when considering the γ fragment in siControl conditions (2.2-fold) or *Fxr1* knockdown conditions (3.4-fold), compared to control empty vector. These data support the hypothesis that FXR1P normally destabilizes *p21* mRNA *via* binding to a motif present in the distal γ portion of its 3′UTR.

To test *in vivo* the hypothesis of FXR1P involvement in the control of endogenous *p21* mRNA stability, we treated siControl- or siFxr1-transfected C2C12 cells with the transcription inhibitor actinomycin D (ActD), and measured the decay rate of *p21* mRNA by quantitative RT-PCR. Interestingly, *p21* mRNA appears to cycle rapidly in control myoblasts. Linear regression on semi-log values of *p21* mRNA decay rate in siControl-transfected cells, provides an estimated half-life of 2.57±0.14 hrs (Figure 7D), with only 16% mRNA remaining after 8 hrs. Conversely, upon *Fxr1*-depletion, *p21* mRNA decay rate is strongly affected and its half-life is significantly increased, reaching 5.98±0.42 hrs (p-val<0.05). As a consequence, even after 8 hrs of ActD treatment, 43% of *p21* mRNA is still present (Figure 7D). The slowing down of *p21* mRNA decay rate following *Fxr1*-knockdown was further confirmed using 5,6-Dichlorobenzimidazole riboside (DRB), an adenosine analogue inhibiting mRNA synthesis (Figure S2). These data suggest that *Fxr1*-depletion increases endogenous *p21* mRNA stability.

### The γ fragment of *p21* 3′UTR contains a highly evolutionarily conserved G-quadruplex motif regulating its stability

**Figure 8:**
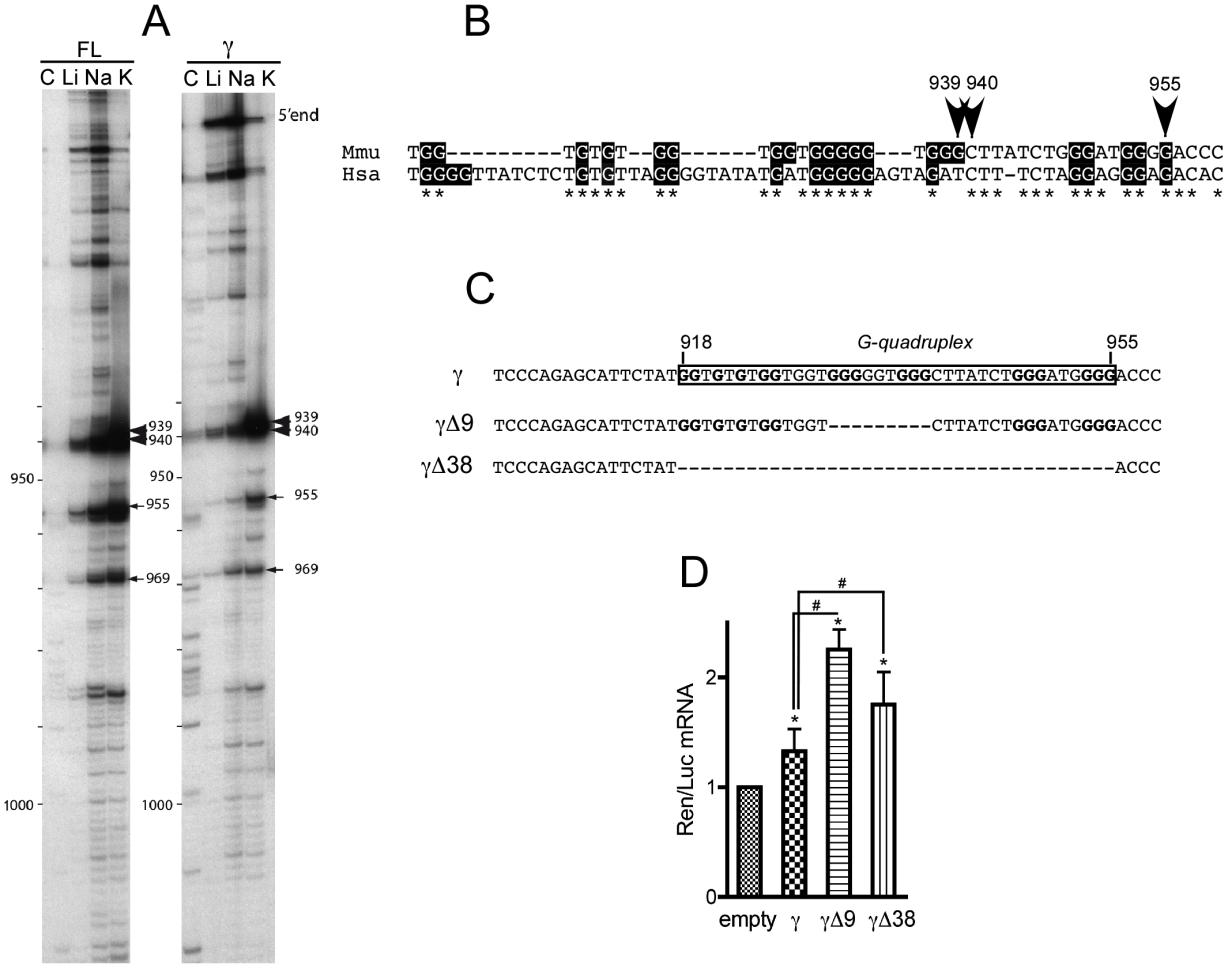
The γ portion of *p21* mRNA 3′UTR contains an evolutionary conserved G-quadruplex structure with mRNA stabilization properties. (A) Cation-dependent termination of reverse transcription in the 3′-UTR full-length (FL) or γ fragment of *p21* mRNA. Strong and weak pauses of reverse transcriptase (RT) are, respectively, indicated by large and thin arrows. Numbers correspond to positions of RT pauses, position +1 being the first nucleotide following the stop codon. (B) Localization and conservation of the G-quadruplex structure detected in (A) on the sequences of *p21* 3′UTR from *Mus musculus* (Mmu) and *Homo sapiens* (Hsa). (C) Scheme of the constructs used for luciferase assays bearing the conserved G-quadruplex of γ *p21* mRNA (boxed) and two versions where the G-quadruplex has been deleted partially (Δ9) and fully (Δ38). (D) Effect of *p21* 3′UTR G-quadruplex and its deletions on *Renilla luciferase* (*Ren*) mRNA stability in C2C12 cells. Quantitative RT-PCR analysis of the levels of *Ren* mRNA normalised to *Firefly* (Luc) mRNA relative to the levels of the empty construct. The γ fragment bearing the G-quadruplex significantly increases *Ren* mRNA levels relative to empty vector. Partial or full deletion of the G-quadruplex sequence strongly increases *Ren* mRNA levels, both relative to empty vector and to the G-quadruplex bearing fragment. The results are presented as the mean of 4 experiments (±SEM). The asterisks * and # indicate p<0.05 respectively of the Wilcoxon test or of the Mann & Whitney test.

The previous data support a negative role for FXR1P in the control of *p21* mRNA stability *via* binding to the 561 nts long *p21* 3′UTR-γ portion. The next step was to determine the RNA motif responsible for FXR1P recognition. So far, two mRNA motifs have been described to be recognized by FXR1P: the ARE motif of *TNFα* mRNA [20] and the G-quadruplex present in *FMR1* mRNA [21]. Our *in vitro* data clearly indicate that the ARE present in the 3′UTR of *p21* mRNA does not mediate the binding of FXR1P Isoe to *p21* mRNA, we therefore looked for the presence of putative G-quadruplex motifs in the γ fragment of *p21* 3′UTR. For this purpose, we used the QGRS webtool [33] that indicated three putative G-quadruplexes spread along the sequence of the γ fragment (Figure S3), and notably a high-score central G-quadruplex motif (nts 931–955). It is worth noticing that this high-score putative G-quadruplex is located within a 51 nts G-rich region (position 918–955, 54% of G). To confirm the predicted G-quadruplex, we used the property of G-quadruplex forming regions to be detected by comparing reverse transcriptase elongation on RNA templates in the presence of either K^+^, Li^+^ or Na^+^ [31]. Indeed, stabilization of G-quadruplex structures by K^+^, but not by Li^+^ or Na^+^, results in cation-dependent pauses detectable on a sequence gel. The experiments were performed on the full-length 3′UTR and on the γ fragment alone and allowed us to identify two strong (position 939 and 940) and two weak G-quadruplex pauses (position 955 and 969) in the 3′UTR of *p21* mRNA (Figure 8A). Both the full-length and the γ fragment exhibited the same pauses, indicating that the γ fragment retains the ability to form the G-quadruplex structure in a comparable manner to the full-length native 3′UTR (Figure 8A). Alignment of sequences corresponding to G-rich regions of *p21* distal 3′UTR in mouse and human indicate high evolutionary conservation of this portion of non-coding sequences (Figure 8B) and argues in favour of its functional importance.

To explore the functional role of the G-quadruplex present in the 3′UTR of *p21* mRNA, we constructed γ fragments mutants with partial (γΔ9) or full (γΔ38) deletion of the G-rich region containing the G-quadruplex (Figure 8C) that were cloned downstream of *Renilla* luciferase mRNA. Then, the levels of *Renilla* mRNA of the resulting constructs were assessed for each mutant in C2C12 cells. As previously shown in Figure 6B, the presence of the γ fragment did increase significantly the levels of *Renilla* mRNA, but partial or full deletion of the G-quadruplex potentiated the increase in the cognate mRNA levels (Figure 8D), mimicking the effect of *Fxr1* knockdown in C2C12 cells (cf Figure 7B). These data argue in favour of a role of the G-quadruplex in mRNA stabilization that is potentiated by deletion of the binding site of FXR1P.

## Discussion

Over the last decade, studies in *Fxr1*-knockout models have inferred that FXR1P plays a critical role in myogenesis [13], [14], [15]. However, even though FXR1P muscle-specific isoforms have unique RNA-binding properties [21], no specific mRNA targets and function have been identified so far for FXR1P in muscle. In this study, we have explored the functional consequences of the depletion of the FXR1P in myoblasts, with the purpose to understand its role in the early stage of myogenesis and in the cellular pathophysiology of FSHD, a human myopathy.

### Cellular pathways affected by *Fxr1*-depletion

Microarray analysis of our myoblastic model inactivated for *Fxr1* enabled to show that FXR1P depletion affects the expression of a wide range of mRNA species that control several cellular pathways. One of the most represented functional categories correspond to ‘skeletal and muscular system development’ and ‘skeletal and muscular disorders’, in line with the evoked role of FXR1P in myogenesis and its altered pattern of expression in two human myopathies: FSHD [9] and DM1 [12]. Interestingly, the functional category ‘cell cycle’ appears also overrepresented in the affected functions, in particular, terms corresponding to ‘arrested in G0/G1 phase transition’ (related to the genes *p21/Cdkn1a*, *HGF*, *IGF*, *IL6*) actually reflect what we observed at the physiological level for *Fxr1* inactivated myoblasts which remain blocked in the G0 phase, without undergoing further differentiation. Apart from *p21*, several mRNAs with altered levels in the absence of FXR1P seem to influence the functional categories affected and appear iteratively in our Ingenuity pathway analysis. These candidates for interaction with FXR1P in the context of myogenesis now deserve further investigation. Notably, *Hepatocyte growth factor* (*Hgf*) mRNA is significantly upregulated in the absence of FXR1P (Table S1, Figure 1E and 1F, Figure S1) and is known to play an essential role in the migration and proliferation of myogenic cells [34]. Similarly, the *Insulin-like growth factor 1* (*Igf1*) would be a relevant target of FXR1P in the muscle context, since Igf1 plays a key regulatory role in skeletal muscle development, as well as muscle fiber regeneration and hypertrophy [35]. Finally, *Cyclin-dependent kinase 15* (*Cdk15*) mRNA which, contrary to *p21* mRNA, is downregulated in *Fxr1-*deficient myoblasts (Table S1, Figure 1E and 1F, Figure S1) would be an interesting candidate for regulation of cell-cycle progression by FXR1P. In this case, FXR1P would stabilize *Cdk15* mRNA via recognition of a yet unknown specific motif. Murine and human *Cdk15* mRNA are not annotated in the AREsite database [36] and therefore do not seem to bear a canonical AU-rich element sequence in their 3′UTR. However, analysis of the 3672 nts long human *Cdk15* mRNA using QGRS G-quadruplex mapping webtool reveals the presence of 8 putative G-quadruplex sequences (Table S3), with 2 putative G-quadruplex in the 3′UTR that represent binding sites for FXR1P. To ascertain the importance of FXR1P in the regulation of its putative mRNA targets newly identified in this study, it would be worth investigating the presence of ARE sequences, G-quadruplexes RNA structures in their 3′ untranslated region.

### Role of FXR1P/G-quadruplex mRNA complex in the destabilization of *p21* mRNA

Adequate regulation of the balance between proliferation and cell cycle arrest of myoblasts is a crucial step during myogenesis. The decision to progress through a new division cycle appears primarily regulated before the G1 to S phase transition, with p21 upregulation playing an important role in this process by blocking the formation of proliferation-inducing Cyclin A/Cdk2-E2F complexes [37]. In this context, *p21* gene undergoes extensive regulation, both at the transcriptional and posttranscriptional level. Our data do not support a transcriptional mechanism for the maintenance of elevated *p21* mRNA levels in *Fxr1*-depleted muscle cells. Indeed, in myoblasts, *p21* is under the sole transcriptional control of the myogenic transcription factor MyoD that activates its promoter [38]. Our microarray and quantitative RT-PCR analyses reveal that MyoD levels remain normal in *Fxr1*-deficient myoblasts (Figure 1E). Finally, in luciferase assays, *Ren* mRNA levels are increased when *p21* mRNA G-quadruplex region is fused to its 3′UTR, even though this mRNA does not contain the endogenous promoter of *p21/Cdkn1a* gene (Figure 7B, Figure 8D). These evidences privilege an FXR1P-mediated posttranscriptional mechanism of regulation of *p21* mRNA levels involving the binding of FXR1P.

In myoblasts, FXR1P long isoforms Isoe and Isof are most likely not playing a role in translational regulation, since they are detected in the nucleus and faintly in the cytoplasm but do not associate to polyribosomes [7], [8], [17], [39]. On the other hand, we cannot exclude a mechanism involving translational inhibition *via* binding of small or medium isoforms of FXR1P to *p21* mRNA to another motif, which may be located in the central part of p21 mRNA 3′UTR (β fragment) that activates translation in the absence of FXR1P (Figure 7A, 7B). This would be consistent with the previously described role of FXR1P small isoform Isoa in translational control [20]. However, our data strongly support the fact that the FXR1P-dependant translational control of *p21* mRNA is mainly regulated by FXR1P long isoforms, notably Isoe, *via* binding to a 3′UTR-located G-quadruplex motif (Figure 8).

To date, the G-quadruplex has been described to be a negative [31], [40] or positive [41] regulator of translation, and a zip-code for dendritic transport and synaptic localization [42] depending on its location on the mRNA (e.g. 5′UTR or 3′UTR) (for review see [43]). We report here an evolutionary conserved G-quadruplex motif as a novel RNA-binding motif present in a G-rich region of the distal portion of *p21* mRNA 3′UTR. This motif, distinct from the classical ARE present in the proximal portion of the 3′UTR [30], appears nevertheless to control the stability of *p21* mRNA. Indeed, when fused to the 3′UTR of *Renilla* luciferase, the G-quadruplex induces an increase in *Renilla* mRNA levels, (Figure 7B, Figure 8D) and this effect is potentiated by deletion of the G-quadruplex (Figure 8D). Collectively, these data argue that the G-quadruplex of *p21* mRNA 3′UTR participates in the control of mRNA stability via a mechanism involving FXR1P. A few reports describe the involvement of 3′UTR-located G-rich stretches as downstream sequence elements (DSE) promoting polyadenylation and leading to increased stability of mRNA when located downstream the polyadenylation site [44], [45]. However, in the context of *p21* mRNA, the G-quadruplex (position 918–955 nts) located upstream of *p21* mRNA polyadenylation site (AAUAAA sequence in position 1309–1314 nts) could act as an upstream sequence elements (USE) promoting polyadenylation, as described for a U-rich sequence in *Prothrombin* mRNA 3′UTR [46]. An alternate mechanism would involve that FXR1P long isoforms drive degradation of *p21* mRNA via recruitment of microRNAs and the RISC complex. RNA interference is well described to occur in the cytoplasm, but it was recently shown that small non-coding RNAs can associate with complementary pre-mRNA target both in the nucleus and in the cytoplasm, by binding to Ago2 [47]. The lattest is a key component of the RNA-Induced Silencing Complex (RISC) [47] and a well-known interactor of FXR1P in human cells [20], *Xenopus* oocytes [48], and in *Drosophila* [49], [50]. Interestingly, *p21* mRNA 3′UTR contains an evolutionarily conserved binding site for miR-22 100 nts upstream of the G-quadruplex motif (Figure S3). This microRNA was recently shown to regulate *p21* mRNA levels [47] and is bound *in vivo* by Ago2 [51]. In this context, *Fxr1*-depletion or *p21* 3′UTR G-quadruplex deletion could prevent recruitment of the RISC complex on *p21* mRNA and contribute to increase its stability, ultimately leading to an accumulation of *p21* mRNA and of the cognate protein.

In myoblasts, FXR1P is not the sole RNA-binding protein playing a key role in the regulation of *p21* mRNA. Several reports demonstrate the importance of the proximal ARE of *p21* mRNA 3′UTR- present in the α fragment- to control the stability of this mRNA. In myoblasts, the ARE-mediated stabilization of *p21* mRNA is mediated by cooperative binding of HuR and hnRNPC1 [30], [52], while its decay is controled by KSRP [53]. Members of the hnRNPE family of proteins, PCBP1 and 2, control the central part of *p21* 3′UTR -the β fragment- [54]. Finally, another hnRNPE, PCBP4, binds and stabilizes the γ fragment [55], while we show in this study that binding of FXR1P to the G-quadruplex motif of p21 3′UTR-γ fragment destabilizes the mRNA. Here, we wish to propose a double system of regulation in which FXR1P and PCBP4 cooperate to regulate the levels of *p21* using the distal 3′UTR while HuR, RNPC1 and KSRP use the ARE in the proximal part. These complex regulatory systems enable a fine-tuning of *p21* mRNA levels, and our data indicate a prominent role for FXR1P as a modulator of *p21* levels.

### FXR1P control of *p21* mRNA stability regulates myoblast cell-cycle exit

We report that, when FXR1P is depleted in the C2C12 cell line and in FSHD myoblasts, p21 levels increase (Figure 1, Figure 4). As a consequence, a subset of myoblasts becomes more permissive to cell cycle arrest, resulting in a reduced yield of myoblasts at each cycle of division (Figure 2, Figure 3). We also observed that the *Cyclin-dependent kinase 15* (*Cdk15*) mRNA levels are decreased (Table S1; Figure 1E and 1F; Figure S1) it would be worth investigating whether its decreased levels also have an impact in this premature cell-cycle exit we observe in *Fxr1*-depleted myoblasts. Our data are in line with other studies in which overexpression of *p21* in myoblasts is sufficient to trigger cell cycle exit, even in mitogenic medium [28], [56], [57]. In our study, *p21* upregulation upon *Fxr1*-depletion causes cell cycle exit without onset of differentiation. Indeed, the levels of the myogenic factors *MyoD* and *Myogenin* remain normal, as assessed by microarray (Table S1) and quantitative RT-PCR (Figure 1F). Moreover, we did not observe spontaneous myoblasts fusion into myotubes in *Fxr1-*knockdown cultures in normal growth conditions, which would be indicative of premature differentiation (Davidovic & Bardoni, unpublished data). Nevertheless, it would be worth investigating in details the impact of *Fxr1*-knockdown on the differentiation of C2C12 myoblasts. Indeed, our data predict that premature cell cycle exit of myoblasts in the absence of FXR1P decreases the pool of myoblasts available for differentiation. This would directly contribute to explain the reduced musculature detected in *Fxr1*-KO mice [13] and in *xfxr1*-knockdown *Xenopus* [14] at early stages of embryogenesis and development.

The fact that *p21* mRNA is an mRNA target for FXR1P Isoe has also crucial implications for the understanding of the pathophysiology of myopathies. Indeed, splicing defects of the *FXR1* gene in FSHD myoblasts leads to reduced expression of the long FXR1P Isoe, the one that specifically binds *p21* 3′UTR. We and others have shown that FSHD myoblasts exhibited higher levels of p21 than controls, under normal growth conditions (this study and [58], [59]). It is now tempting to speculate that depletion in FXR1P Isoe directly participates to the physiopathology of FSHD, by causing p21-mediated premature arrest of the cell cycle in FSHD myoblasts. Ultimately, this may limit the pool of myoblasts available for regeneration of muscle fibers, inducing progressive muscle wasting in FSHD patients. This hypothesis is supported by a study which demonstrates that p21 is essential for normal myogenic progenitor cell function in regenerating skeletal muscle [60]. A similar scenario may be envisioned in the case of the mouse model of DM1 in which reduced expression of FXR1P Isoe was determined [12].

### Conclusions

In conclusion, our study highlights for the first time the direct involvement of an RNA-binding protein, FXR1P, in a new pathway that regulates *p21* levels to control myoblasts cell cycle exit. Perturbations of this pathway will have a strong impact in muscle development and implicates a new signal dependant on a 3′-UTR located G-quadruplex-RNA structure. In the future it will be important to explore the implication of FXR1P in pathophysiology of muscle disorders and the pleiotropic functions of FXR1P during myogenesis. Furthermore, our study opens new perspectives on the role of the other Fragile X related proteins in the control of cell cycle. Noteworthy, FMRP is known to recognize G-quadruplex mRNA structures and it would be tempting to speculate that FMRP could control *p21*-dependant cell cycle exit of neuronal progenitors during neurogenesis.

## Materials and Methods

### Cells

The C2C12 cell line, a subclone of the C2C4 murine myoblastic cell line [61], [62], was cultivated under confluence state in the conditions described by ATCC. C2C12 cells were transfected with siRNA targeting exon 14 or exon 6 of *Fxr1* mRNA (see Table S1) and/or constructs using the Lipofectamine 2000 reagent (Invitrogen), according to the manufacturer's protocole. Control experiments were performed using commercially available control random siRNA of matching GC content (Invitrogen). Transfected cells were always analysed 48 hrs post transfection. mRNA decay experiments were performed by adding actinomycin D (Act D, 5 µg/mL) or 5,6-Dichlorobenzimidazole riboside (DRB, 50 µM) to culture medium for 0 to 8 hrs.

Human myoblasts derived from muscle biopsies of n = 3 FSHD patients and n = 3 controls of matching age and gender were described in [9]. The procedures to generate myoblasts derived from human muscle biopsies were agreed by the French Health Authorities (AFSSAPS). Myoblasts cultures were established as previously described [9].

### Gene expression profiling

Total RNA of C2C12 cells transfected with siFxr1 or siControl siRNAs was extracted using the RNeasy kit (Qiagen, Hilden, Germany). Integrity of RNA was assessed by using an Agilent BioAnalyser 2100 (Agilent Technologies) (RIN above 8). RNA samples were then labeled with Cy3 dye using the low RNA input QuickAmp kit (Agilent) as recommended by the supplier. 825 ng of labeled cRNA probe were hybridized on 8×60K high density SurePrint G3 gene expression mouse Agilent microarrays. Two biological replicates were performed for each experimental condition. The experimental data are deposited in the NCBI Gene Expression Omnibus (GEO) (http://www.ncbi.nlm.nih.gov/geo/) under the series record number GSE40577. Normalization of microarray data was performed using the Limma package available from Bioconductor (http://www.bioconductor.org). Inter slide normalization was performed using the quantile methods. Means of ratios from all comparisons were calculated and B test analysis was performed. Differentially expressed genes were selected based on a B-value above 0. Data from expression microarrays were analyzed for enrichment in biological themes (Gene Ontology molecular function and canonical pathways) and build biological networks using Ingenuity Pathway Analysis software (http://www.ingenuity.com/) and Mediante (http://www.microarray.fr:8080/merge/index), an information system providing information about probes and data sets.

### Quantitative RT–PCR

Total RNA was extracted from myoblasts using the RNeasy kit (Qiagen, Hilden, Germany) and a reverse transcription (RT) reaction was performed using the Superscript II RT-PCR system (Invitrogen, Carlsbad, California, USA) according to the manufacturers' protocol. RT products were subjected to polymerase chain reaction (PCR). All primers were designed using the Primer 3 software (Table S4). Standard RT-PCR was performed using the Promega PCR Master Kit (Promega, Madison, Wisconsin, USA). Real-time PCR reactions were carried out using the Syber Green I qPCR core Kit (Eurogentec, Liège, Belgium) in a LightCycler system (Roche, USA). The comparative threshold cycle (C_t_) for the amplicons of each sample was determined by the LightCycler software and normalised to the corresponding C_t_ of *TATA Box Binding Protein* (TBP) mRNA for endogenous *p21* mRNA levels, and to the C_t_ of *Firefly* luciferase in the case of *Renilla* luciferase mRNA assessment. Finally, the 2-ΔΔC_t_ method [63] was used to analyse the relative changes in the various studied mRNAs between C2C12 myoblasts transfected with control siRNA (Invitrogen) or anti-*Fxr1* siRNA (Invitrogen), or between FSHD myoblasts and controls (n = 3). Data were expressed as means ±SEM. Each assay was performed in triplicate with n = 3–4 independent replicates.

### Immunoblot and immunofluorescence

Cell extracts were analysed by western blotting as described previously [64], [65]. Previously described primary antibodies against FXR1P were polyclonal rabbit antibody #830 (1∶5,000) and monoclonal 3FX (1∶500), the latter also cross-reacting with FXR2P [7]. Anti-β-actin monoclonal antibody (Sigma) and anti-p21 polyclonal rabbit antibodies (Santa Cruz) were used respectively at 1∶10,000 and 1∶200. Digital acquisition of chemiluminescent signal was performed using the Las-3000 Imager system (Fujifilm). Quantitation of western-blot was performed using the ImageJ software and normalized to the β-actin signal.

Immunofluorescence was performed as described [9], using anti-FXR1P #830 polyclonal antibodies (1∶5,000; [8]) and anti-Ki67 monoclonal antibody (1∶100; Millipore). Secondary Alexa 594-coupled antibodies (Invitrogen, Carlsbad, California, USA) were used at 1∶250. After counterstaining with DAPI, coverslips were mounted on slides with anti-fading reagent and observed using a Zeiss Axioplan2 epifluorescence microscope equipped with a CoolSNAP HQ CCD cooled camera (Roper Scientific) or an Olympus FV10i confocal digital microscope. Micrographs were then analysed with ImageJ software.

### Cell viability and FACS analysis

For viability assessment 48 hrs post transfection with anti-*Fxr1* and control siRNAs, both attached cells and culture supernatant were collected and then incubated in the presence of propidium iodide (PI, 50 µg/mL). The incorporation of PI in dead cells was then analysed with a FACScan instrument (Becton, Dickinson). MTT proliferation assay was used to determine the proliferation ability of the cells as recommended by the manufacturer (Sigma). For cell cycle distribution assessment, cells were fixed in 70% ethanol, treated with RNAseA (50 µg/mL), stained with PI (50 µg/mL) and their DNA content was assessed using FACS analysis. For synchronisation experiments, cells were treated with 500 nM of the cell blocker mimosine for 8 hrs. Release from cell cycle blockade was performed for 16 hrs in growth medium before FACS analysis.

### RNA–binding assays

Human FXR1P Isoe recombinant protein His-tagged in the C–terminus was produced in bacteria using the pET21a/FXR1 Isoe construct [21], as described [64]. The control RNA fragments used in this study: N19 (RNA sequence derived from *FMR1* mRNA and containing a G-quadruplex forming structure) and N19Δ35 (N19 sequence in which the G-quadruplex is deleted) were cloned in pTL1 plasmid [31]. The various fragments from *p21* cDNA were amplified by RT-PCR of C2C12 cDNAs and cloned in the pGemTEasy system (Promega) using the primers described in Table S1, as advised by the manufacturer. For filter binding assay, N19 or p21 constructs were *in vitro* transcribed using T7 RNA polymerase (Promega), the RNA products being labeled by cotranscriptional incorporation of [γ^−32^ P]-ATP. Labeled RNAs were purified on a 1% low-melting agarose gel (Ambion). Labeled RNAs (50,000 c.p.m., 4 fmol) were renatured for 10 min at 40°C in binding buffer (50 mM Tris–HCl (pH 7.4), 1 mM MgCl_2_, 1 mM EDTA, 150 mM KCl, 1 mM DTT). In the presence of 2 U/mL of RNase inhibitors (RNasin, Invitrogen), 0,1 mg/mL of *Escherichia coli* total tRNA and 0.01% BSA, radiolabeled RNA were incubated to increasing amounts of FXR1P protein. RNA–protein complexes were allowed to form for 10 min on ice, filtered through MF-membranes (0.45 HA, Millipore) and washed with 2 mL binding buffer. Filters were air-dried and Cerenkov counting was used to assess the levels of remaining radioactivity on filters. Data were plotted as percentage of total RNA bound versus the protein concentration and one-site binding curve was drawn using the Prism 4 software.

### UV-crosslinking and immunoprecipitation (CLIP)

To isolate mRNAs associated with FXR1P *in vivo*, UV-crosslinking and immunoprecipitations (CLIP) were performed with extracts of C2C12 cells using a protocol adapted from [65] and the #830 polyclonal antibody directed against the C-terminus of FXR1P [8]. For each assay, 10 µg of polyclonal antiserum was used to immunoprecipitate 25×10^6^ cells. An equivalent amount of unrelated rabbit IgGs (Sigma) were used as negative control. Approximately 1/20th of the homogenate and 1/4th of the immunoprecipitate were loaded on a 11% SDS–PAGE gel. Proteins transferred onto a 0.45 µm nitro-cellulose membrane were revealed using the 3FX antibody recognizing both FXR1P and FXR2P [8]. mRNAs were extracted from C2C12 input lysate and immunoprecipitates using Trizol reagent (Invitrogen) according to the manufacturer's protocole and subjected to reverse transcription (RT) using the SuperscriptScript III RT-PCR system (Invitrogen). RT products were subjected to polymerase chain reaction (PCR), using a PCR Master Kit (Promega) and primers detailed in Table S4 specific for *p21*, *Myogenin*, *MyoD* and *β-Tubulin* mouse cDNAs. The PCR program consisted in 10 min. of initial denaturation at 95°C followed by 35 cycles −30 s. at 95°C, 30 s. at 58°C, 30 s. at 72°C- and a final elongation step of 10 min at 72°C. PCR products were visualised on a 2% TAE agarose gel and amplicon size was verified using the 1 Kb+ DNA ladder (Invitrogen).

### Luciferase assays

Luciferase assays were performed using the pSiCheck2 system (Promega) according to the manufacturer's protocole. Briefly, the various fragments from *p21*-3′UTR cDNA (α, β and γ) were excised from the pGemTEasy vectors using the NotI site and inserted downstream of the *Renilla* luciferase cDNA using the NotI site of the pSiCheck2 vector. C2C12 cells were co-transfected in 96-well plates with the siRNA control or against *Fxr1* and pSiCheck2 constructs. Luciferase assays were performed 48 hrs post transfection using the DualGlow Luciferase Kit (Promega) according to the manufacturer's protocole.

### Constructs

pTL1/FXR1Isoe plasmid was cloned as described in [8]. The mutated version of this plasmid bearing 4 silent mutations in human *FXR1* cDNA that impede recognition by siFxr1#1 was produced by site-directed mutagenesis using primers described in Table S4 and the QuickChange kit (Stratagene).

### Statistical analysis

To compare numerical data, non-parametric Mann & Whitney test was used for small sample size (n<30) and a Student T-test was used when n>30. Wilcoxon non-parametric tests were used to assess significance of *Renilla* luciferase mRNA or activity levels variations between each fragment relative to the empty vector (arbitrarily set to 1). All statistical analysis and data graphs were performed with the Prism 4 software. Only significant differences are displayed on the graphs.

## Supporting Information

Figure S1 Confirmation of microarray mRNA candidates using a second siRNA targeting another constitutive exon of *Fxr1* mRNA (exon 6). (A) Quantitative RT-PCR reveals a strong reduction of *Fxr1* mRNA in C2C12 cells transfected with siRNA against *Fxr1* (siFxr1#2) compared to siControl-transfected cells. (B) Western-blot analysis of untransfected (UT) and siFxr1-transfected cells (siFxr1#2) revealed with the antibody #3FX recognizing all isoforms of FXR1P reveals a strong depletion of all isoforms of FXR1P (short, medium and long) compared to control (siCtl), while the levels of FXR2P protein (asterisk, *) remain unchanged. β-tubulin (β-tub) signal is used to verify equal loading of lanes. (C) Quantitative-RT PCR analysis of a subset of mRNAs confirm that *Sema7a*, *Mctp2*, *Asrg1*, *Cdkn1a/p21*, *Hgf*, *Dusp6* and *Lbh* mRNAs are significantly upregulated while *Cdk15* mRNA is downregulated in *Fxr1*-depleted C2C12 myoblasts, confirming the microarray analysis and quantitative-RT PCR analysis using the first siFxr1 siRNA. Data are presented as means ± SEM of n = 4 experiments.
(TIF)

Figure S2 Confirmation that *Fxr1*-depletion increases the stability of endogenous *p21* mRNA using a second transcription inhibitor. C2C12 transfected with siControl (empty squares) or siFxr1 (black squares) were treated with the transcription inhibitor 5,6-Dichlorobenzimidazole riboside (DRB) for 8 hrs. Percentages of remaining *p21* mRNA at the various time points were determined by quantitative RT-PCR and normalised to levels before treatment (t0). During the first 5 hrs of treatment, *p21* mRNA stability is clearly increased when FXR1P is knocked-down by siFxr1 transfection, as compared to siControl-transfected cells. At the dose used, DRB effect is reversible and transcription resumes after 5 hrs of treatment resulting in a progressive increase in *p21* mRNA levels in both conditions.
(TIF)

Figure S3 Sequence analysis of *p21* 3′UTR γ fragment bound by FXR1P in search for G-quadruplexes and microRNA binding sites. The position and scores of the three putative G-quadruplexes structures predicted by the webtool QGRS [33] in the γ portion of murine *p21* mRNA 3′UTR are boxed. The putative G-quadruplex located between nts 931–955 displays a high score of 38 and lies within a G-rich region (nts 918–968) in which G are highlighted by empty circles (°). A conserved binding site for miR-22/22-3p conserved among species is located in position 837–843 nts, as predicted by TargetScan webtool.
(TIF)

Table S1 List of the 79 RefSeq annotated transcripts significantly modulated after *Fxr1*-depletion in myoblasts. NCBI RefSeq IDs give access to transcripts annotations. Logarithm (base 2) of the average intensity (AveExp) and logarithm (base 2) of the ratio siFxr1/siControl are represented. The subset of mRNAs further validated in quantitative RT-PCR are highlighted in bold.
(XLSX)

Table S2 Ingenuity Pathway Analysis of microarray data to highlight selectively affected pathways in *Fxr1*-depleted myoblasts. All the affected pathways ordered by p-value are presented in (A), while pathways specifically related to ‘skeletal muscle’ or ‘cell cycle’ are respectively presented in (B) and (C).
(XLSX)

Table S3 Prediction of G-quadruplexes present in human *Cdk15* mRNA using QGRS webtool. G-quadruplexes displaying the highest scores are localized in the 5′UTR or coding sequence of the mRNA.
(XLSX)

Table S4 List of primers used in study.
(XLSX)
