# Supplementary figures and images for: A Novel Role for the RNA–Binding Protein FXR1P in Myoblasts Cell-Cycle Progression by Modulating p21/Cdkn1a/Cip1/Waf1 mRNA Stability
Source: PLoS Genet. 2013 Mar 21;9(3):e1003367. doi: 10.1371/journal.pgen.1003367 (PMC3605292; doi:10.1371/journal.pgen.1003367)

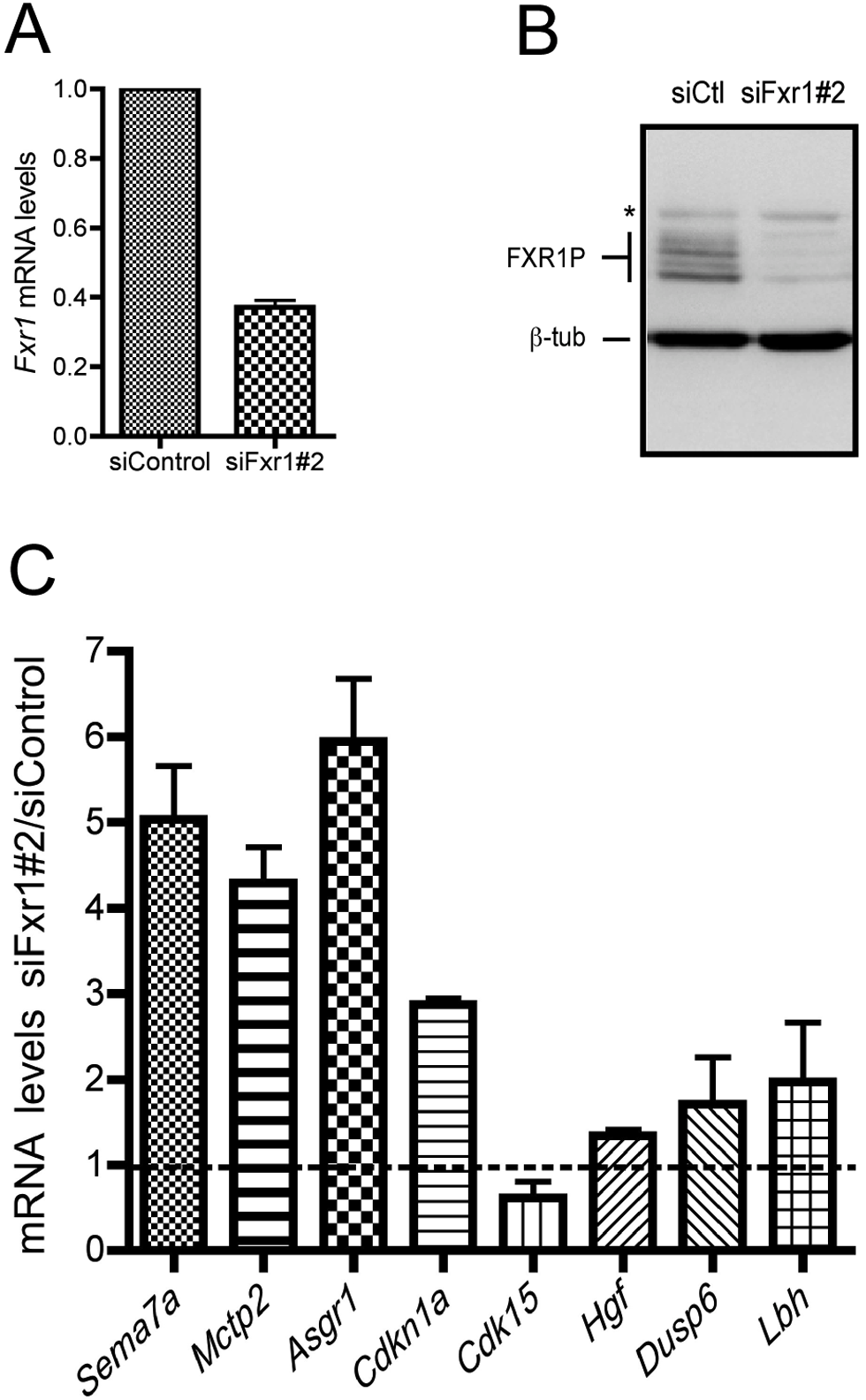

Supplement: Figure S1 — Confirmation of microarray mRNA candidates using a second siRNA targeting another constitutive exon of Fxr1 mRNA (exon 6). (A) Quantitative RT-PCR reveals a strong reduction of Fxr1 mRNA in C2C12 cells transfected with siRNA against Fxr1 (siFxr1#2) compared to siControl-transfected cells. (B) Western-blot analysis of untransfected (UT) and siFxr1-transfected cells (siFxr1#2) revealed with the antibody #3FX recognizing all isoforms of FXR1P reveals a strong depletion of all isoforms of FXR1P (short, medium and long) compared to control (siCtl), while the levels of FXR2P protein (asterisk, *) remain unchanged. β-tubulin (β-tub) signal is used to verify equal loading of lanes. (C) Quantitative-RT PCR analysis of a subset of mRNAs confirm that Sema7a, Mctp2, Asrg1, Cdkn1a/p21, Hgf, Dusp6 and Lbh mRNAs are significantly upregulated while Cdk15 mRNA is downregulated in Fxr1-depleted C2C12 myoblasts, confirming the microarray analysis and quantitative-RT PCR analysis using the first siFxr1 siRNA. Data are presented as means ± SEM of n = 4 experiments. (TIF) [file pgen.1003367.s001.tif]

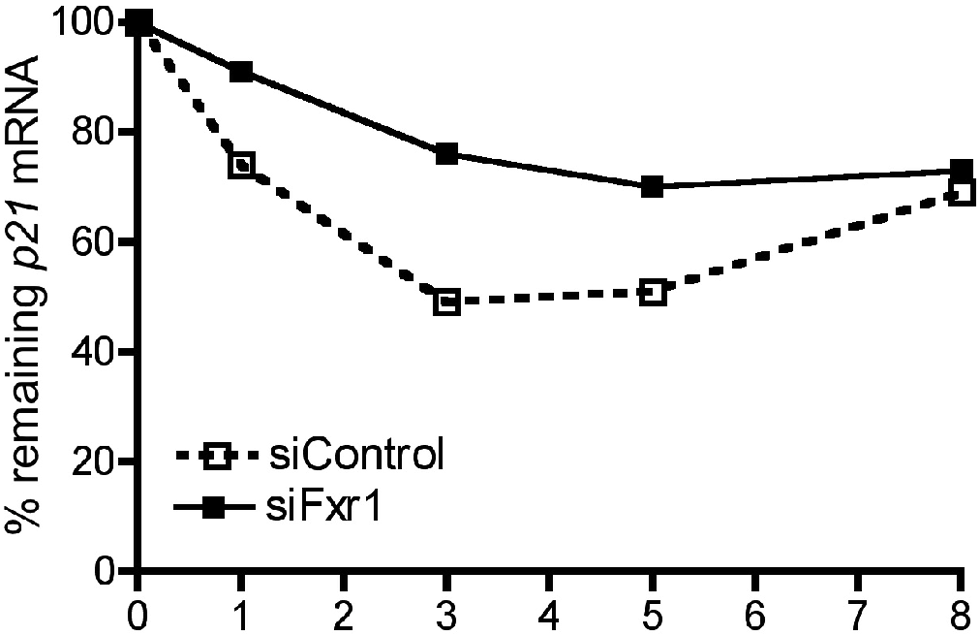

Supplement: Figure S2 — Confirmation that Fxr1-depletion increases the stability of endogenous p21 mRNA using a second transcription inhibitor. C2C12 transfected with siControl (empty squares) or siFxr1 (black squares) were treated with the transcription inhibitor 5,6-Dichlorobenzimidazole riboside (DRB) for 8 hrs. Percentages of remaining p21 mRNA at the various time points were determined by quantitative RT-PCR and normalised to levels before treatment (t0). During the first 5 hrs of treatment, p21 mRNA stability is clearly increased when FXR1P is knocked-down by siFxr1 transfection, as compared to siControl-transfected cells. At the dose used, DRB effect is reversible and transcription resumes after 5 hrs of treatment resulting in a progressive increase in p21 mRNA levels in both conditions. (TIF) [file pgen.1003367.s002.tif]

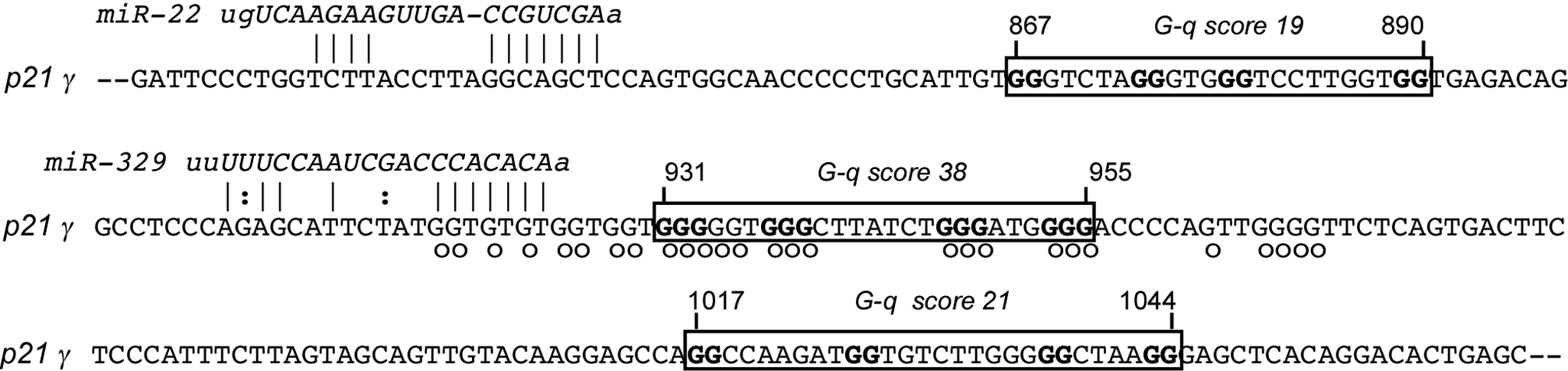

Supplement: Figure S3 — Sequence analysis of p21 3′UTR γ fragment bound by FXR1P in search for G-quadruplexes and microRNA binding sites. The position and scores of the three putative G-quadruplexes structures predicted by the webtool QGRS [33] in the γ portion of murine p21 mRNA 3′UTR are boxed. The putative G-quadruplex located between nts 931–955 displays a high score of 38 and lies within a G-rich region (nts 918–968) in which G are highlighted by empty circles (°). A conserved binding site for miR-22/22-3p conserved among species is located in position 837–843 nts, as predicted by TargetScan webtool. (TIF) [file pgen.1003367.s003.tif]
